# Application of Neuroscience Tools in Building Construction – An Interdisciplinary Analysis

**DOI:** 10.3389/fnins.2022.895666

**Published:** 2022-06-21

**Authors:** Mengmeng Wang, Xiaodan Liu, Yu Lai, Wenna Cao, Zhiyong Wu, Xiaotong Guo

**Affiliations:** ^1^School of Management, Xi’an University of Architecture and Technology, Xi’an, China; ^2^Laboratory of Neuromanagement in Engineering, Xi’an University of Architecture and Technology, Xi’an, China

**Keywords:** building construction, neuroscience tools, bibliometric, physiological tools, neurophysiological tools

## Abstract

Interdisciplinary integration is a new driving force in development of science and technology. Neuroscience, a powerful tool for studying human physiology and psychology that is greatly interconnected with the field of building construction, has attracted numerous research attention. In this paper, we systematically review the interdisciplinary applications of neuroscience tools using bibliometric methods. We report that the built environment, construction safety, architectural design, and occupational health are the main areas of research attention, while thermal comfort, air quality, hazard recognition, safety training, aesthetic design, and biophilic design, among others, comprise the most frequently studied topics with regards to application of neuroscience tools. Currently, eye tracking and the electroencephalogram are the most commonly used tools in the field of building construction, while functional near-infrared spectroscopy, functional magnetic resonance imaging and trigeminal nerve stimulation are still at their initial stage of application.

## Introduction

With the global education movement flourishing in the 21st century, cultivation of interdisciplinary thinking in institutions of higher learning has received widespread attention worldwide (Aliyeva, 2022). For example, China established the 14th interdisciplinary subject in January 2021 to vigorously promote the country’s interdisciplinary development. Interdisciplinary is seen as the key to addressing complex societal challenges, with the integration of knowledge across multiple disciplines playing a crucial role in generation of effective solutions because of the increasing complexity and cross-disciplinarily of current challenges across industries (O’Donovan et al., 2021). Therefore, in the Era of Big Science, new scientific knowledge and discoveries are often generated by the intersection among different disciplines (Cockburn, 2021). Moreover, scientific research and technological advancement are increasingly dependent on such cross-disciplines. For instance, Sun et al. (2021) believed that the interpenetration, interaction, and integration of knowledge between different disciplines generate innovative new knowledge and technologies. Consequently, interdisciplinary research has not only become an important growth point for knowledge innovation and development, but also an important driving force for determining national science and technology innovation capacity. In addition, it is a current mainstream form of research, as evidenced by various disciplines that have attempted to apply knowledge from other disciplines, such as theories, methods and tools, to solve complex problems within themselves.

Neuroscience is a frontier discipline that applies biological mechanisms to explore human cognitive functions and mental activities. Its technical tools have received numerous attention from various disciplines, such as economics, marketing, and education, among others (Alvino et al., 2020). Consequently, neuroscience tools have become an excellent choice for interdisciplinary research, due to their ability to capture data directly from the human body to complement existing data sources. For example, these tools allow researchers to measure human response data directly as people are engaged in various activities (e.g., decision making) or in response to various stimuli (Dimoka et al., 2012), a phenomenon that has now been extended to the field of architecture. The concepts, methods and tools of architecture, a traditional industry, have remained unchanged for quite some time. However, the field of building has increasingly focused on human issues, owing to the increase in building standards and usage requirements (Wang et al., 2021), such as safety cognition and mental workload of workers during construction (Chen et al., 2016) as well as environmental perception of indoor personnel during usage (Kühn et al., 2021). Since these issues cannot be solved by the knowledge of building science alone, there is need for interdisciplinary integration between architecture and neuroscience, with support from architecture, neuroscience tools and psychology-related knowledge (Eberhard, 2009).

Neuroscience tools can not only measure stimuli-induced physiological signals, but also real-time activity signals of the brain. Boz et al. (2017) and Stasi et al. (2018) broadly classified neuroscience tools into two categories, namely physiological and neurophysiological tools. Particularly, physiological tools allows one to measure both voluntary and involuntary reflexes, such as fixating and tracking visual stimuli, whereas neurophysiological tools can record and analyze brain activity thereby allowing researchers to study human psychology and behavior, such as electroencephalogram (EEG). Currently, scholars have reviewed the interdisciplinary application of neuroscience tools in the field of building construction. For example, Zhang et al. (2019) and Saedi et al. (2022) summarized the application of EEG in the field of building construction safety, and analyzed the frequency bands of EEG as well as the channels used to detect the electrical activity of the brain. On the other hand, Cheng et al. (2022a) reviewed the application of eye tracking techniques in construction safety, and described the different index used to study human mental performance in visual, cognitive and attention aspects. Moreover, Hu and Shealy (2019) explored the application of functional near-infrared spectroscopy (fNIRS) in engineering decision making and design cognition, by exploiting the relationship between brain and behavior in engineering settings.

Although neuroscience tools have gradually been applied in building construction, and review articles highlighted the application of specific tools such as EEG and eye tracking, only a handful of studies have comprehensively reviewed the applications of neuroscience tools in building construction from a general standpoint. Moreover, little is known regarding the structure of interdisciplinary integration between neuroscience tools and building construction. In view of the interdisciplinary field of neuroscience tools applied to building construction research, this paper adopts the basic statistical methods in bibliometric to analyze the trend in development of this interdisciplinary field. Particularly, we first explored the most influential countries, institutions, scholars and journals in this field, trying to reveal the whole picture of the knowledge development of neuroscience tools in building construction domain from a macro perspective. Next, we applied the co-word analysis method in bibliometric, and established the co-occurrence network of keywords in this interdisciplinary field as well as cluster distribution of keywords based on the co-occurrence relationship, with the aim of generating knowledge on this interdisciplinary field from a micro perspective. Overall, this paper provides a comprehensive and objective panorama of knowledge development for scholars who seek to understand this interdisciplinary field, to provide latest research information and hot and emerging frontier knowledge topics. Moreover, we provide a valuable literature reference for scholars in the fields of environmental science, public health, sustainability, and neuroscience related to building construction.

## Materials and Methods

### Data Retrieval

We first determined search terms for literature retrieval. To this end, we divided neuroscience tools into two main categories, namely physiological and neurophysiological tools. When discussing application of neuroscience tools in the field of marketing and information systems, Dimoka et al. (2012) and Alvino et al. (2020) reported the use of various physiological tools, including electrocardiography (ECG), electromyography (EMG), electrodermal activity (EDA), eye tracking (ET), and voice pitch analysis (VPA), among others, as well as neurophysiological tools such as electroencephalogram (EEG), functional magnetic resonance imaging (fMRI), functional near-infrared spectroscopy (fNIRs), magnetoencephalogaphy (MEG), transcranial magnetic stimulation (TMS), and transcranial direct current stimulation (tDCS), among others. Numerous measurement tools are currently applied in neuroscience. However, searching these tools in the database and limiting them to the field of building construction, after searching one by one, revealed that only a handful of them have actually been used for research in the field of building construction. Only nine neuroscience tools have been so far used in this field (Table 1). The search terms used in this study are also outlined in Table 1.

**TABLE 1:** Search terms for neuroscience tools used in building construction.

Tool classification	Search terms
Physiological tools	Electrocardiogram (ECG)
	Electromyography; electromyogram (EMG)
	Electrodermal activity (EDA); galvanic skin response (GSR); Skin conductance responses (SCR); skin conductance level (SCL)
	eye tracking; eye tracker (ET)
Neurophysiological tools	Electroencephalogram; electroencephalography; electro-encephalography (EEG)
	event related potential*(ERP)
	functional near-infrared spectroscopy (fNIRS)
	functional magnetic resonance imaging (fMRI)
	Trigeminal nerve stimulation (TNS)

**FIGURE 1:**
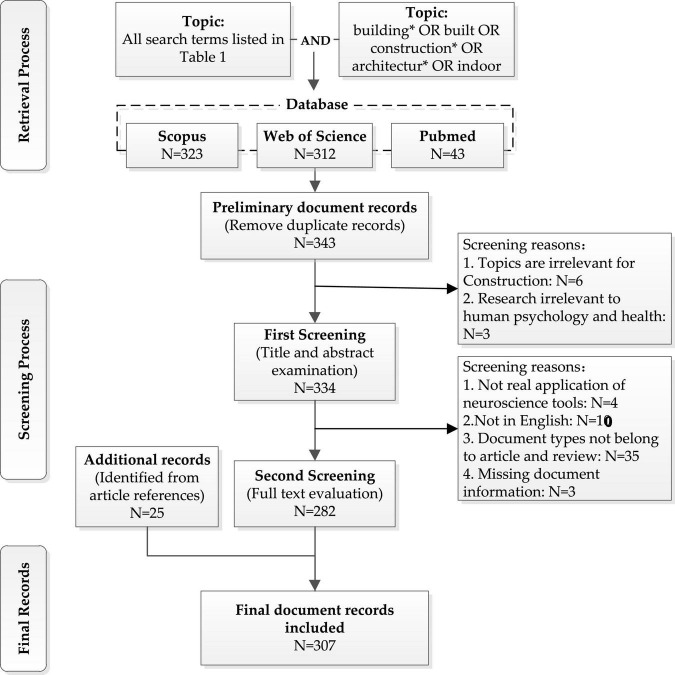
Flowchart describing the procedure for document retrieval and screening.

After identifying the search terms for each neuroscience tool, we searched the Web of Science Core Collection, Scopus, and PubMed databases for articles in the field of building construction, which applied neuroscience tools. Among them, Web of Science and Scopus cover literature in the fields of natural sciences, social sciences and other scientific fields, while PubMed focuses on literature in the medical field. We focused on these three commonly used databases to collect complete literature in this interdisciplinary field. The specific search information is shown in Supplementary Table 1. We also screened the bibliographies in the initially downloaded articles, as shown in Figure 1, to identify further literature. Then, we adopted the method by Zhang et al. (2019) to remove duplicate and merging documents across the three databases. To this end, two manual screenings were used to identify relevant and eligible articles. Finally, a total of 307 articles were included in the study. Details from the articles, including the title, author, abstract, keywords, source publication, country, institution, publication year, references, and citation frequency, were retrieved.

### Methodology

Bibliometric is a discipline that uses mathematical methods to count the results of scientific research, describe the structure of science, analyze the inner workings of scientific systems, and explore the quantitative laws of scientific activity as a whole (Wang et al., 2022). In this study, we explored the application of neuroscience tools in building construction from an interdisciplinary standpoint, mainly using basic statistical and co-word analysis methods in bibliometric. Co-word analysis, which combines bibliometric and text mining techniques, is a content analysis method that mines the deep semantic relationships between terms. Since it uses co-occurrence patterns of words and phrases in a corpus, it can establish relationships between ideas and concepts in the subject domain thereby presenting them in the corpus. Specifically, if two or more keywords co-occur in a literature, these keywords are considered to have a co-occurrence relationship with each other. Co-word analysis identifies the strength of co-occurrence between terms and creates a set of lexical maps that effectively illustrate the strongest associations between individual terms. In the current Information Age, co-word analysis has made it easier for researchers to extract knowledge from texts, including research and conference papers, as well as newspaper articles and book chapters, thus allowing them to identify the degree of semantic closeness between each other *via* frequency of occurrence between specific keywords in the text.

**FIGURE 2:**
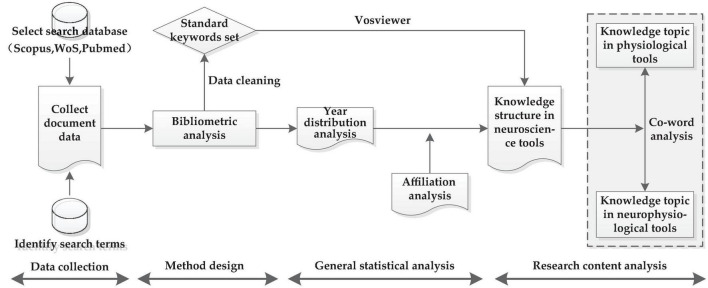
Research framework of neuroscience tools used in building construction.

The research framework of this paper is shown in Figure 2. Based on the literature data in this interdisciplinary field, we applied the bibliometric method targeting two aspects of research. The first involved the use basic statistics to analyze the distribution of numbers and citations in literatures to mine the law of change in this field. This method was also used to analyze the affiliation distribution of literatures, mine the most influential countries and institutions, as well as the most authoritative scholars and journals in this field, in order to generate the whole picture of the development of the field. The second aspect entailed the use keywords as the carrier of knowledge content. Since the same meaning of keywords may have different variants, expressions or abbreviations, after cleaning the keyword data, a standard keyword set is constructed. Then, the co-occurrence function in Vosviewer software was used to cluster the keywords, identify the knowledge structure of this interdisciplinary field from the perspective of content analysis, and further mine the core knowledge topics in the application research of each neuroscience tools in the field of building construction, according to co-occurrence frequency of the keywords.

## Overview of Application of Neuroscience Tools in Building Construction

### Yearly Distribution of Publications

**FIGURE 3:**
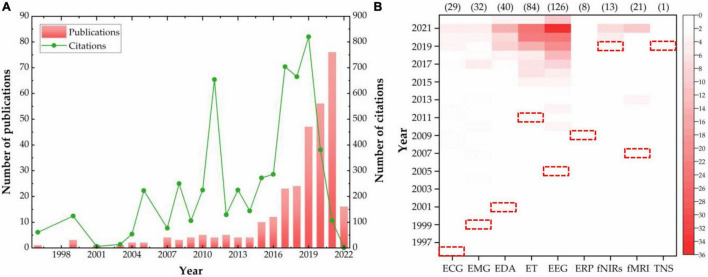
Yearly distribution of publications. **(A)** Distribution of publications and citations. **(B)** Distribution of tools in neuroscience.

Literature in this interdisciplinary field were subjected to quantitative statistics and results presented in a graph shown in Figure 3A. Summarily, the first article in this field appeared in 1996. The number of studies has been rapidly rising since 2010, reaching a peak of 76 articles in 2021. The overall trend is close to an exponential growth pattern, in line with the natural law of scientific knowledge growth discovered by Price (1975). Analysis of citations over the years revealed that the overall citations have also grown steadily, albeit with small fluctuations. The highest number of citations was 820 in 2019, followed by 704 in 2017, and indicated that neuroscience has been getting more and more attention from scholars in the field of building construction. Distribution of the number of literatures in the application research of a single neuroscience tool over the years is depicted using a heat map in Figure 3B. Notably, the red dotted box represents the time when each tool was first applied in the field of building construction. Summarily, ECG had cross-applications with the building construction field as early as 1996, while fNIRS and TNS were applied in this field at the latest in 2019. According to literature trends in the heat map, the two neuroscience tools, ET and EEG, had the most application, which rose steadily each year. Therefore, ET and EEG are the mainstream neuroscience tools used in the current research process of related issues in the field of building construction.

### Affiliation Analysis of Publications

**FIGURE 4:**
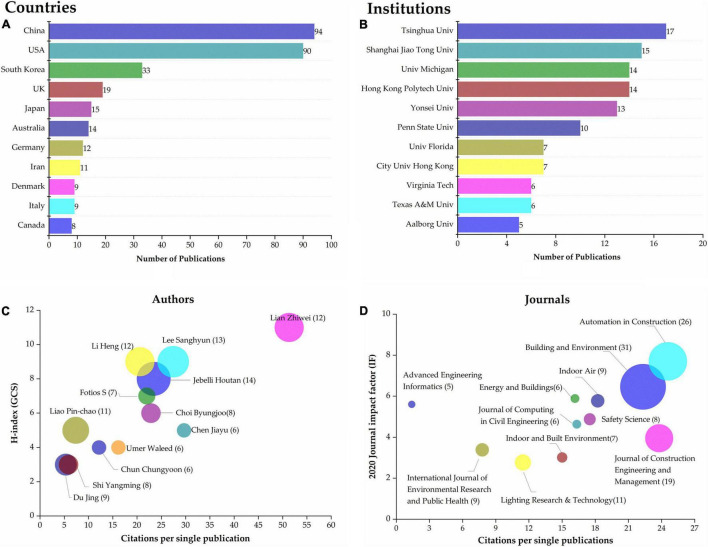
Country analysis **(A)**, institution analysis **(B)**, author analysis **(C)**, and journal analysis **(D)** of publication in this study.

The distribution profiles of retrieved articles across countries, institutions, journals, and authors are shown in Figure 4. Figure 4A shows the top eleven countries with 8 or more articles, among which the highest number of articles were from China and the United States, followed by the United Kingdom and South Korea. This indicated that these countries have the most extensive research with regards to application of neuroscience tools. Meanwhile, analytical results of research institutions from which these articles were published are presented in Figure 4B. Summarily, Tsinghua University in China had the highest number of articles, followed by Shanghai Jiao Tong University. Notably, 4 of the eleven institutions with the highest number of articles were from China, with 5 of them from the United States, further affirming that these two countries are the main research sites for the application of neuroscience tools in building construction. Profiles of the most influential scholars are presented in Figure 4C. Summarily, Jebelli Houtan from University of Michigan, United States, has the highest number of publications, while Lian Zhiwei from Shanghai Jiao Tong University has the highest average citation and H-index. It is worth noting that the difference between the H-index and the number of publications of scholars Lian Zhiwei, Fotios S, and Chen Jiayu was only within 1, indicating these scholars’ articles are generally of high quality. Distribution of core journals to which these articles belong is illustrated in Figure 4D. From the data, it is evident that Building and Environment has the highest number of publications, while Automation in Construction has the highest average citation. The impact factor of these journals ranged between 2.5 and 8, which indicates that the application of neuroscience tools has gained numerous attention and recognition across the mainstream academic community in the field of building construction.

## Knowledge Fusion of Neuroscience Tools in Building Construction

### Structure Analysis in the Application of Neuroscience Tools

**FIGURE 5:**
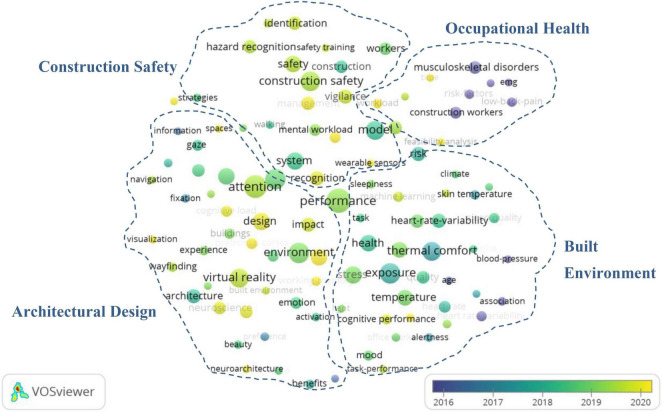
Keywords co-occurrence network of neuroscience tools in building construction.

Keyword collection of literatures published in a field can reveal the content characteristics of the research. Therefore, by counting the frequency of keywords in a certain field, we can understand the research focus of the field to a certain extent. Co-word analysis can be used to reveal the co-occurrence relationship between keywords. Notably, appearance of two keywords in the same article reveals that there is a co-occurrence relationship between the two keywords, a phenomenon that reflects the content correlation between keywords. A stronger co-occurrence relationship implies a higher knowledge correlation between the content reflected by the keywords. Vosviewer is a commonly used co-word analysis software, which can establish a co-word network based on the co-occurrence relationship of keywords. Since a stronger co-occurrence denotes a closer distance between keywords, different clusters can be formed according to the distance, thereby reflecting different knowledge content. Based on this, we employed Vosviewer to mine the knowledge structure of this interdisciplinary field. Firstly, we imported the standardized keyword data into the software and selected Co-occurrence analysis, then selected keywords with a frequency of six or more. Finally, we generated a co-word network as shown in Figure 5.

According to the figure, the size and color of the nodes represents the frequency of an average year of keywords, respectively, while the distance between nodes indicates the co-occurrence relationship among keywords. It is worth noting that nodes with high co-occurrence frequency are closer to each other. Therefore, safety, environment, and thermal comfort, among others, are the key knowledge topics for the application of neuroscience tools in the field of building construction. On the other hand, hazard recognition, wayfinding, and cognitive load, among others are the emerging topics in the field of building construction that have applied neuroscience tools in recent years. Analysis of the clustering formed by the co-occurrence relationship indicated that application of neuroscience tools to the building construction field is concentrated around four sub-domains, as follows:

#### Built Environment

Urban dwellers spend 80–90% of their time indoors, a phenomenon that exacerbates the impact of the built environment on the human body. Indoor built environment generates a microclimate that creates a certain temperature, humidity, light and other conditions, integrating various physical phenomena interacting with the space, while the human body has a dynamically physiological and psychological stress response to changes in the indoor environment, affecting the comfort perception and satisfaction with the indoor environment. Previous studies have reported application of current neuroscience tools to various aspects of building indoor environment research, including exploration of thermal comfort, sleep quality, and cognitive performance under different indoor conditions of temperature, humidity, and airflow (Pan et al., 2012; Lan et al., 2019; Lang et al., 2022). These tools have also been employed to uncover the effects of high concentrations of ozone, carbon dioxide, aerosols, particulate matter and other air pollution on human physiology, neurophysiology as well as other physical and mental health aspects (Huang et al., 2019; Snow et al., 2019). Moreover, the tools have been instrumental in identifying the effects of different light conditions, light sources, and lighting tools on human visual comfort, sleep, attention, alertness, and mood, among others (Lin and Westland, 2020). Furthermore, studies have described the use of these tools to successfully identify the effects of different types and levels of noise on human physiological and psychological responses (Lan et al., 2021). In addition, neuroscience tools have also been applied in research on indoor and outdoor plants. For example, scholars explored the effects of indoor plants on human comfort, mental stress, happiness as well as other physiological and psychological effects (Hassan et al., 2020; Elsadek and Liu, 2021), and the effects of different plants and colors in outdoor green spaces and the layout of green landscape on mental health (Huang et al., 2021).

#### Construction Safety

The high number of injuries and accidents in the construction industry has been attributed to long-term exposure of construction workers to risks coupled with little sensitivity to hazards. Previous studies have shown that accurate and timely identification of accident hazards is imperative to effective reduction of accidents at construction site and maintenance of construction safety (Wang et al., 2017). Traditional safety assessment methods, such as interviews and questionnaires, have been previously used. In recent years, neuroscience tools have been gradually applied as a more immediate and objective method. Construction hazard recognition is the most frequently applied topic. EEG can be applied to measure the level of physical and mental fatigue among construction workers and assess the safety hazards, such as trips, falls, and impacts, caused by workers’ mental and emotional state including depression, and stress, thereby improving health and productivity (Hasanzadeh et al., 2018; Tehrani et al., 2021). Scholars have also employed eye tracking to explore the visual search and attention allocation patterns of workers with different personality traits, knowledge levels, and experience levels (Fu et al., 2022). This approach has enabled effectively identification of on-site hazards, and analysis of the probability of hazards caused by search defects (Hasanzadeh et al., 2017). Accordingly, risk perception is a key part of workers’ safety decision making. Therefore, active safety training for construction workers is imperative to improving their risk alertness, attention levels and risk perception (Choi et al., 2019). Neuroscience tools, targeting neuroscience measurement, are also commonly used to study workers’ attitudes and willingness to train safely, assess the degree of improvement in safety hazard recognition ability under different training modes, and establish post-training safety warning as well as job performance assessment systems (Comu et al., 2021).

#### Architectural Design

Architectural designs should consider both the aesthetic and functional requirements of the building. Currently, neuroscience tools are frequently used in architectural design, mainly with regards to developing architectural aesthetic, environmental and interior navigation designs. Architectural appearance greatly impacts the aesthetic experience of the observer, while the judgment of beauty and unattractiveness are closely related to personal expertise as well as subjective feelings. Therefore, architects usually use eye tracking technology to not only determine the aesthetic effect of architectural elements but also explore the relationship between the observer’s gaze pattern with the formal properties of architectural elements under different design parameters (Jam et al., 2021). Previous studies have also reported the use of ERP and fMRI in analysis of key psychological dimensions sensitive to specific design parameters and the neural features evoked (Ma et al., 2015; Coburn et al., 2020). For interior environment design, a combination of virtual reality (VR) with EEG/ERP, ET has been used to effectively measure neurophysiological information related to human subjective feelings as well as cognitive functions across different interior environments. These include the proportion of interior functional areas, lighting designs, room color schemes, furniture color and other spatial environment designs, with respect to human emotions, security and comfort (Tuszynska-Bogucka et al., 2020). In terms of navigation design, since indoor wayfinding is a daily and complex activity, current researches have also applied high-resolution, immersive virtual reality (VR) technology, in combination with EEG and ET, to mine human way finding data. Consequently, these data have enabled effective understanding of the working memory and cognitive workload during execution of navigation tasks by designing different route parameters in the virtual platform, and finally propose the optimal route plan for interior design (Vecchiato et al., 2015).

#### Occupational Health

The construction industry is one of the most labor-intensive industries. Notably, workers in this sector are faced with demanding physical tasks almost every day, which take a toll on their health, as evidenced by prevalence of various conditions such as musculoskeletal disorders. For example, Umer et al. (2017) reported that steelworkers suffer from low back pain caused by prolonged static or awkward postures, while Anton et al. (2013) demonstrated that porters suffer from shoulder pain and other bodily pain when carrying various types of loads. These tasks expose workers to musculoskeletal disorders caused by a variety of ergonomic risks. Current EMG techniques in neuroscience have been applied in analysis of occupational health hazards among construction workers, such as in identification of the degree of chronic muscle fatigue, an important cause of musculoskeletal disorders (Seo et al., 2016). Particularly, this has been achieved by continuously monitoring biomechanical variables, including trunk muscle activity and trunk kinematics through surface electromyography and motion sensing (Umer et al., 2017), thereby determining which tasks cause excessive physical strain on construction workers. To reduce the risk of musculoskeletal disorders among the workers, construction managers need to better understand the physical and biomechanical requirements of various construction tasks. This will allow them to not only effectively implement appropriate preventive measures and improve occupational health in the construction industry, but also increase productivity of the construction workers.

### Topic Analysis in the Application of Physiological Tools

#### Electrocardiogram

The electrocardiogram (ECG) is a non-invasive technique for measuring the electrical activity and electrophysiological response of the heart. Its simplicity, portability, and good temporal resolution make it ideal for clinical use. Heart rate variability (HRV) is a physiological measure of the variability of the human heart rate cycle and contains information on the regulation of the cardiovascular system by neurohumoral factors, reflecting the tension and balance of cardiac sympathetic and parasympathetic activity and their effect on cardiovascular system activity. HRV level is considered to be an indicator of physiological stress or arousal, increasing with low HRV and decreasing with high HRV. Therefore, changes in HRV can be used to analyze information about human thoughts, emotions, and behaviors, revealing individuals’ psychological responses to different environments or stimuli, such as emotional, fatigue, stress, and other psychological states. Table 2 details the most commonly used ECG analysis indexes in the field of building construction. By recording various HRV indexes, information on the autonomic nervous system and stress state can be acquired to provide clues and a basis for the study of human factors in this field.

**TABLE 2:** Physiological parameters measured by ECG.

Parameter		Implication
Time-domain analysis parameters	SDNN	Sensitive indicators for assessing sympathetic nerve function
	RMSSD	Sensitive indicators for assessing parasympathetic function.
	pNN50	Reflects the tension level of the parasympathetic or vagal nerve
Frequency domain analysis parameters	HF	Reflects parasympathetic or vagal activity
	LF	Reflect sympathetic nerve activity
	LF/HF	Reflects the balanced control of the autonomic nervous system

**FIGURE 6:**
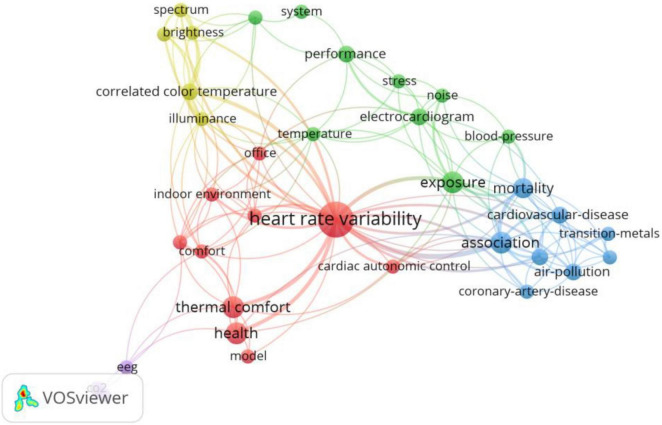
Keywords co-occurrence network of ECG in building construction.

Figure 6 shows the co-word network of ECG used in the field of building construction. As illustrated in the figure, ECG is mainly used in research topics related to the indoor environment. Among the thermal comfort topics, research has revealed that cold indoor temperatures have a negative effect on cardiovascular health (Umishio et al., 2021), whereas human thermoregulation is related to thermal comfort and is regulated by the autonomic nervous system. HRV is sensitive to changes in ambient temperature and human thermal sensation and may be utilized as a potential physiological indicator of human thermal comfort (Liu et al., 2008). When thermal sensation is neutral or slightly significant, sympathetic and parasympathetic nerves are in relative equilibrium. When thermal sensation is significant or thermal discomfort is present, sympathetic nerves are in a more active state and can trigger emotions such as stress and anxiety (Yang et al., 2021). Within the context of air pollution, studies have demonstrated that higher levels of indoor exposure to carbon monoxide (Riojas-Rodriguez et al., 2006), carbon dioxide (Zhang et al., 2021), and ozone (Huang et al., 2019) result in elevated HRV, as reflected by decreased parasympathetic regulation (RMSSD, HF, pNN50) and increased sympathetic drive (SDNN, LF, LF/HF), while increased exposure to indoor particle pollutants, especially PM2.5, causes HRV levels to decrease (Cavallari et al., 2007), thus increasing cardiovascular risk (Jia et al., 2012). Within the context of indoor plants, it is demonstrated that the LF/HF ratio of viewing plants of different colors is significantly negatively correlated to human satisfaction, demonstrating that HRV is a valid physiological parameter for assessing the comfort provided by indoor plants (Qin et al., 2014).

#### Electromyogram

Electromyography (EMG) is a technique that utilizes surface electrodes to acquire bioelectrical signals generated during neuromuscular system activity. EMG signal analysis focuses on both time-domain analysis and frequency domain analysis. The purpose of this study is to investigate the possible causes of EMG changes and reflected muscle activity and function by correlating the time domain and frequency domain characteristics of EMG and muscle structure, as well as muscle activity and functional status. In recent years, EMG has also been gradually used in the construction field. Researchers typically connect EMG sensors to the upper arm, cervical spine, thoracic spine, lumbar spine, and back of workers to monitor their muscular status during heavy lifting, repetitive weight lifting, awkward kneeling, or squatting postures, and prolonged knee and neck flexion, etc. The most often used metrics for analyzing the EMG signals include, root mean square normalization, mean absolute value, median frequency, etc. (Ahn et al., 2019).

**FIGURE 7:**
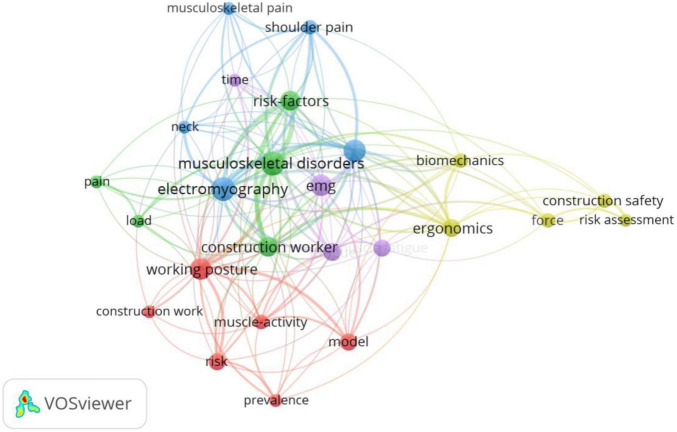
Keywords co-occurrence network of EMG in building construction.

Figure 7 shows the co-word network of EMG used in the field of building construction. As can be observed, the use of EMG in the building construction field is mostly focused on the identification of muscle fatigue and the analysis of construction workers’ muscle ergonomics. In the area of muscle fatigue recognition, the amplitude of the EMG increases with the degree of fatigue during the process of muscle isometric contraction to fatigue, and studies have shown that the fatigue rate of the lumbar muscles of steelworkers is significantly higher than other parts of the body (Antwi-Afari et al., 2017) and that the EMG activity of lumbar muscles decreases significantly when bending over to assemble rebar (Umer et al., 2018). When construction workers are working on the roof, the slope of the roof and their kneeling posture have a significant effect on the peak activation of knee muscles (Dutta et al., 2020). Additionally, EMG can also detect the development of muscle strength and fatigue of construction workers in performing repetitive manual tasks and can predict the degree of muscle fatigue by analyzing the root mean square normalized amplitude of EMG (Li et al., 2017). Muscle ergonomics analysis demonstrates that the use of passive exoskeleton systems significantly reduces lumbar vertical spine muscle activity and that the reduction is even greater when weight lifting loads (Antwi-Afari et al., 2021). Meanwhile, EMG measurements of upper body muscle load between painters of different sexes reveal that female house painter have a higher relative muscle load than their male counterparts (Meyland et al., 2014).

#### Electrodermal Activity

Electrodermal activity (EDA) is a physiological technique for determining the conductivity of human skin by applying a small but constant voltage to the skin and measuring changes in electrical currents caused by sweat secretion (Mansi et al., 2021), including skin conductance level (SCL) and skin conductance response (SCR), where SCL indicates sympathetic activity induced by chronic stress and SCR can explain the correlation between specific stimulus events and levels of emotional arousal. Because sweat glands are innervated by the sympathetic nervous system, EDA is an ideal measure of sympathetic activation, which is influenced by the hypothalamus and limbic system (brain regions associated with emotion), making EDA a good indicator of an individual’s mood, arousal, stress, attention, and risk perception levels (Mansi et al., 2021). EDA is currently the most effective and sensitive physiological parameter for detecting changes in individual sympathetic arousal due to its high stability, ease of measurement, and high sensitivity (Mansi et al., 2021).

**FIGURE 8:**
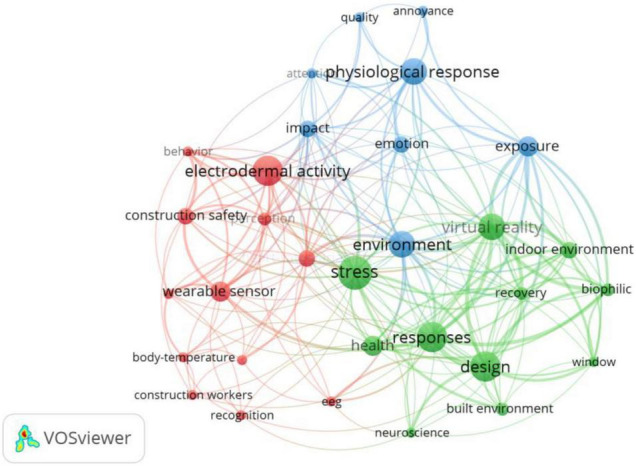
Keywords co-occurrence network of EDA in building construction.

Figure 8 shows the co-word network of EDA used in the field of building construction. At the moment, EDA is primarily used in the built environment, where excessively high or excessively low indoor temperature (Chou et al., 2016; Sepehri et al., 2021), and a cold white lighting environment (Basso, 2001) can increase EDA, thereby increasing work stress for indoor workers. When people spend an extended period in a noisy environment, their respiration rate drops sharply and their EDA levels significantly increase (Park et al., 2018). Indoor placement of plants and artificial windows reduces people’s SCL levels, demonstrating that natural elements can be effective in relieving stress and reducing arousal (Kim et al., 2018), and similarly installing green walls indoors helps alleviate negative emotions (Yin et al., 2018). Wearable biosensors with integrated EDA capabilities are used in construction safety studies to obtain physiological signals from workers to assess their stress levels and the physical demands required for different jobs (Jebelli et al., 2019). For example, studies have found that workers with loads produce higher EDA values than those without loads (Anwer et al., 2021), and EDA can also be used to predict concentration levels by monitoring workers’ biosignals (Kim et al., 2021). EDA is often used in conjunction with VR in architectural design research to investigate the perception of human experience and emotional responses in architectural spaces and built environments (Ergan et al., 2019). For example, SCR may be used to measure and quantify the effect of geometric manipulation of building spaces on human emotional responses (Shemesh et al., 2021), as well as the effect of irregular exterior window shapes and sunlight exposure conditions on occupant pleasantness in the interior (Chamilothori et al., 2019).

#### Eye-Tracking

Eye-tracking (ET) is a technique that measures the properties of human eye movements as they process perspective information and is commonly used in studies on attention, visual perception, etc. Eye movement is closely related to visual attention, which is typically associated with cognitive processing, which determines human behavior. Consequently, human behavior can be efficiently analyzed by monitoring eye movement trajectories. The primary measures used in ET research include gaze and saccade, as well as a variety of derived metrics based on basic measurements such as gaze, scan path, pupil size, blink rate, etc. Similarly, ET can also define areas of interest (AOI) based on the scene and measure eye fixation and saccade between AOIs, including the number of fixations and running counts within the AOI. Increased fixation time and count of fixations to a specific area indicate increased interest in the target. With the evolution of ET technology, eye trackers are becoming increasingly prevalent in building construction, where two distinct types of eye tracker systems are commonly used. One is a stationary eye tracker system that requires the subject to sit in front of a monitor for visual tasks, while the other is a mobile eye tracker that uses an eyeglass-like sensor to record eye movement data as well as changes in head position (Hasanzadeh et al., 2018).

**FIGURE 9:**
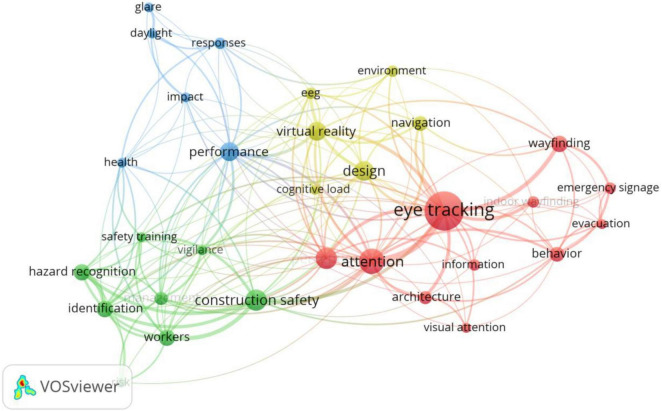
Keywords co-occurrence network of ET in building construction.

Figure 9 illustrates an ET co-word network used for research in the field of building construction. Since ET is widely used in construction, this section further details research on the core knowledge topics listed in Table 3. ET is mostly used in the built environment to study the light environment, where glare is a significant factor affecting the visual comfort of indoor personnel. PUI, BA, FR, and other indicators can indicate visual discomfort caused by glare, but increasing ambient luminance contrast can improve efficiency in a non-glare environment (Liu et al., 2021). In construction safety, it is discovered that the visual search patterns of workers who correctly identify hazards differ from those who do not, with attention deficit and mental fatigue being the primary reasons for workers’ decreased ability to detect hazards. The decrease is related to a change in the distribution of fixation and gaze points, and the use of wearable eye-tracking equipment can help identify construction hazards more accurately. From a safety training perspective, scan paths and fixation heat maps generated by ET technology can effectively reveal focused or personalized feedback to workers, and this feedback communicates to workers defects in the search process, eliciting reflection and thereby facilitating hazard recognition (Jeelani et al., 2018). ET technology has been used in architectural design to assess the aesthetic effect of architectural elements based on the gaze pattern of the eyes and has also been applied to the perception of building interior and exterior decoration and geometric design, reflecting people’s emotional response to various architectural spaces through pupil diameter and fixation time. ET is frequently used in conjunction with VR technology in indoor wayfinding studies to examine indoor navigation and signage design using metrics such as fixation time. Additionally, biophilic design elements such as greenery are gaining popularity, and the use of eye movement metrics such as total fixation time can help designers better understand how office design can contribute to occupant’s health and performance (Guo et al., 2022).

**TABLE 3:** Research summary of ET applications in building construction.

Topic	Subtopic	Index	Eye tracker	References
Built environment	Light environment	Mean Pupil Diameter (PD), Pupillary Unrest Index (PUI), Blink Rate (BR), Blink Amplitude (BA), eye Fixation Rate (FR), and Eye Convergence (EC)	Tobii Pro Glasses 2	Garreton et al., 2015; Hamedani et al., 2020; Liu et al., 2021
Construction safety	Hazard recognition	Fixation count, fixation duration, time to first fixation, pupil size, fixation heat map, scan paths, intersection coefficient	Tobii Pro Glasses 2, Tobii T60 XL	Sun and Liao, 2019; Xu et al., 2019; Han et al., 2020; Cheng et al., 2021; Chong et al., 2021; Wang et al., 2021
	Attention	Fixation time, fixation count, run count, dwell time, run count, dwell percentage, and first fixation time, Pupil dilation, Saccadic velocity, Saccadic duration	Tobii Pro Glasses 2, EyeLink II, Tobii Glasses II	Hasanzadeh et al., 2017; Hasanzadeh et al., 2018
	Mental fatigue	Blink rate/count, blink duration, pupil diameter, percent change in pupil diameter (PCPD), fixation duration, fixation count, gaze point position	Pupil Labs	Li et al., 2019; Li et al., 2020; Cheng et al., 2022a
	Safety Training	Dwell Time, Fixation Counts (FC), Fixation Time (FT), Mean Fixation Duration (MFD), Visual Attention Index (VAI), Ratio of On-Target: All-target Fixation Time (ROAFT), Time to First Fixation	Tobii Glasses 2, Tobii Pro X2-30 Hz, SMI iView XTM HED	Jeelani et al., 2018; Wang et al., 2018; Comu et al., 2021
Architectural design	Aesthetic design	Fixation Duration	Tobii Pro Glasses 2, EyeLink	Azemati et al., 2020; Jam et al., 2021
	Architectural perception	Mean fixation duration, fixation count, Pupil Diameter, time to first fixation, number of fixations during observation, average number of fixations, total duration of all fixations, number of visits during observation, average number of visits per person	Tobii TX300, Pupil Labs ET, Tobii X2-30	Tuszynska-Bogucka et al., 2020; Shemesh et al., 2021
	Indoor Navigation	Relative number of fixations, fixation counts, fixation time	Tobii Pro Glasses 2	Schrom-Feiertag et al., 2017
	Biophilic design	Total Fixation Duration, fixation time	Tobii Glasses 2, Tobii Pro VR Integration	Yin et al., 2019; Lei et al., 2021

### Topic Analysis in the Application of Neurophysiological Tools

#### Electroencephalogram and Event-Related Potential

The electroencephalogram (EEG) is a technique for recording brain activity using electrophysiological indicators. It records the electrical activity generated by neurons in the cerebral cortex by amplifying microscopic electrical signals in the cerebral cortex. The human brain is made up of tens of thousands of neurons that transmit information that causes the voltage across its membranes to vary in milliseconds, resulting in brain waves of different frequencies. Each rhythm of brain waves is associated with a specific state of the brain, as shown in Table 4, and these rhythms are identified by frequency and amplitude. The event-related potential (ERP) is a type of brain evoked potential, that is formed by the synchronization of postsynaptic potentials of a large number of neurons induced by a stimulus event, and it reflects neurophysiological changes in the brain during cognitive processes (Hou et al., 2021). EEG and ERP have high temporal resolution and can identify human emotions, cognition, and other psychological conditions. They are being used more widely used in the field of building construction than other methods. Table 5 shows the building topics analyzed and the EEG indicators involved.

**TABLE 4:** Specific information on EEG frequency bands.

Frequency bands	Frequency	Mental state	Research topic
Delta (δ)	0.5–3.5 Hz	Delta appears in deep non-REM sleep and is usually located in the thalamus	sleep disorders, alcoholism
Theta (θ)	4–7 Hz	Theta is associated with difficulties with mental operations (e.g., inattention, distraction, memory difficulties, anxiety, depression, etc.)	Mental load, working memory, cognitive effort, anxious temperament
Alpha (α)	8–12 Hz	Alpha occurs when emotions are stable or relaxed, is associated with relaxed wakefulness, and contributes to mental coordination, calmness, and alertness	physical and mental relaxation
Beta (β)	13–30 Hz	Beta occurs during moments of mental activity, busy or anxious thinking, and is associated with focus, analysis, conscious alertness	Concentration, stress levels, alertness levels
Gamma (γ)	> 30 Hz	Gamma occurs in advanced information processing or complex mental activities such as cognition, memory or associative learning	Cognitive processing, problem solving, learning, facing cognitive challenges

**TABLE 5:** Research summary of EEG applications in building construction.

Topic	Subtopic	Index	Channels	Mental State	References
Thermal environment	Thermal comfort	α power, β power, γ power, power density spectra (PDS), Mental workload index	Cz, C3, C4, Fz, F3, F4, P3, POz, AF3, AF4, T7, T8, Pz	Sleep quality, cognitive load, mental workload, working memory, perception, reaction, attention	Yao et al., 2009; Zhang et al., 2017; Choi et al., 2019; Wang et al., 2019
	Thermal pleasure	Relative β power, relative θ power	P3, P4, Cz, C3, C4, Fz, F3	Emotion, pleasant, satisfaction, relaxation	Son and Chun, 2018; Han and Chun, 2021
Acoustic environment	Noise	θ/β power, Asymmetry index (ASI) of α power	AF3, AF4, F3, F4, F7, F8, FC5, FC6, C3, C4	Attention, stress, mental workload	Ke et al., 2021; Lan et al., 2021
Lighting environment	Illuminance, color temperature	β power, frontal asymmetry index (FAI), α-band percentage	FP1, FP2, C3, C4, O1, O2, F3, F4	Sleep, relaxation, work engagement	Kakitsuba, 2016; Deng et al., 2021
Air quality	Air pollution	α relative power, β relative power, High-δ power, θ relative power	C3, Cz, C4, P3, P4, P7, P8, Pz, T7, T8, O1, O2, FC6, F8	Executive function, reaction time, working memory, attention, cognitive flexibility	Shan et al., 2019; Snow et al., 2019; Zhang et al., 2021
Plant environment	Indoor plant	Relative EEG power	C3, CZ, F3, FZ	Comfort, attention, memory	Qin et al., 2014; Elsadek and Liu, 2021; Llinares et al., 2021
Construction safty	Mental Workload	Power spectral densities (PSD), EEG-engagement index (EN)	TP9, FP1, FP2, TP10	mental workload, inattentional blindness	Chen et al., 2016, 2017, 2022
	Hazard Recognition	PSD, average value of amplitude	14 channels	Hazard perceptions	Jeon and Cai, 2021; Noghabaei et al., 2021
	Fatigue Monitoring	(θ + α)/β, (θ + α)/(α + β), α/β, θ/β	14 channels	Drowsiness, mental fatigue	Aryal et al., 2017; Li et al., 2019; Xing et al., 2020
	Attention and vigilance	α power, PSD, relative EEG power	14 channels	*Attention, vigilance, distraction*	Ke et al., 2021; Wang et al., 2017, 2019; Cheng et al., 2022b
	Stress recognition	α, β, θ, δ mean power, Median frequency, PSD	AF3, AF4, F3, F4, FC5, FC6, F7, F8, P7, P8, O1, O2	Stress	Jebelli et al., 2018; Chae et al., 2021
	Emotional state	PSD	14 Channel	Emotion	Hwang et al., 2018; Xing et al., 2019
Architectural design	Space design	Ratio of α to β waves (RAB), power spectrum, event-related spectral perturbations (ERSPs)	F4, P3, F7, CP2, FC6, P3	Emotion, stress and anxiety, relaxation, arousal	Banaei et al., 2017; Ergan et al., 2019
	Environmental design	PSD, Individual α frequency (IAF)	F3, P7, Pz, P4, P8, O1, O2, FP2, F8	Novelty, comfort, pleasantness, arousal	Vecchiato et al., 2015

**FIGURE 10:**
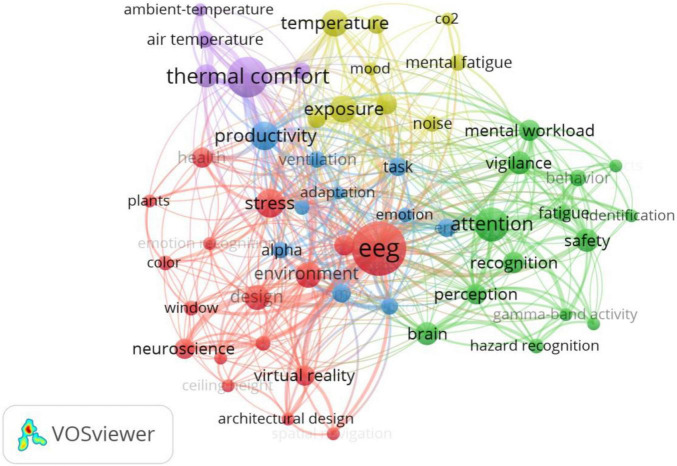
Keywords co-occurrence network of EEG/ERP in building construction.

On the basis of the co-word network of EEG in Figure 10, Table 5 further summarizes the research topics and sub-topics of EEG applied to building construction, the commonly used EEG indicators, and EEG channels, the cognitive and emotional states, and the representative references. Thermal perception in the built environment is determined by the arousal and mood of the indoor thermal environment, and temperature changes affect brain rhythms and the power spectral densities (PSD) of brain electricity, which affects thermal comfort and thermal pleasure. Construction noise, as well as prolonged indoor noise in the building sound environment, can affect people’s minds and bodies. The alpha band which is excited by varying degrees of illumination and color temperatures in the lighting environment can reflect people’s mental states. When indoor air quality is poor and pollution levels are high, theta and beta powers of the human brain increase and can result in bad moods, whereas the alpha waves of the human brain are more active when viewing indoor plants and can significantly relieve personal stress. When workers are subjected to different stressors during construction, different brain wave patterns are generated, reflecting their emotional state, attention level, mental workload, and other mental activities. Monitoring and identification of worker fatigue using EEG can significantly reduce construction hazards, while low-frequency gamma waves can also reflect workers’ vigilance and attention levels. According to research, theta waves in the anterior cingulate cortex are associated with the emotional perception of specific geometric landscapes. EEG can help improve the architectural design and create environments that meet human needs by better understanding the impact of architectural design on human perception and subjective experience. Furthermore, when summarizing the existing literature in the field of building construction applied EEG, the analysis of data extracted by EEG primarily uses machine learning methods such as artificial neural network (ANN), support vector machine (SVM), random forest (RF), and hidden Markov models (HMM).

#### Functional Magnetic Resonance Imaging

Functional magnetic resonance imaging (fMRI) is a non-invasive imaging technique commonly used to study brain function. Its basic principle is to use magnetic resonance imaging to measure the hemodynamic changes induced by neuronal activity. While fMRI offers some advantages such as non-invasiveness, reproducibility, high spatial resolution, etc., its temporal resolution is low compared with neurophysiological tools such as EEG and fNIRs. Therefore, fMRI can dynamically track the changes of signals in various brain regions, such as those induced by thinking activities and cognitive experiences. Additionally, the high-resolution brain imaging images generated by fMRI are better visualized, making the technique more accessible to non-expert audiences. However, fMRI also has limitations in terms of motion restrictions and higher costs, mainly due to the cost of maintaining the equipment and hiring technicians to operate the scanner.

**FIGURE 11:**
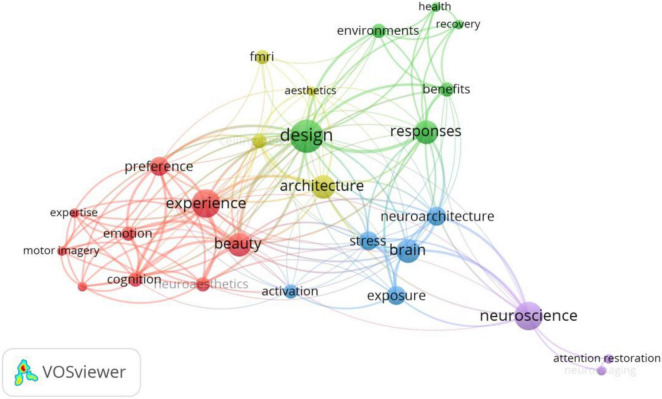
Keywords co-occurrence network of fMRI.

The co-word network in Figure 11 shows the application research for fMRI in the field of building construction. As illustrated in the figure, fMRI is mainly used in studies on architectural design. Because architecture’s aesthetic quality affects people’s well-being, the aesthetic response to architecture can be explained in terms of psychological dimensions associated with specific neural features of the brain (Coburn et al., 2020). Scholars have discovered that the prefrontal and hippocampal brain areas are involved in the evaluation of architectural aesthetics, with the primary factor affecting the activation of the parahippocampus place area being the openness of the space, which is typically regarded as more beautiful (Chatterjee et al., 2021). By studying the effect of ceiling height on aesthetic judgments in architectural design, researchers discovered that visuospatial processing activates the left anterior cuneiform and left middle frontal gyrus of the brain (Vartanian et al., 2015). Specific architectural styles can also induce contemplative states; for example, certain contemplative buildings (e.g., museums, churches, libraries, etc.) cause significant activation of the left posterior central gyrus and left inferior parietal phase, eliciting contemplative experiences (Bermudez et al., 2017). Additionally, construction noise has been studied using fMRI, and it was discovered that persistent noise exposure may alter the limbic gyrus (particularly the cingulate and parahippocampal gyrus), as well as adhesion locations, including the anterior cuneus and posterior lobe of the cerebellum (Dai and Lian, 2018).

#### Functional Near-Infrared Spectroscopy

Functional near-infrared spectroscopy (fNIRS) is a wearable spectroscopy system that measures blood oxygen concentration to detect neurocognitive activation. fNIRS works by emitting near-infrared light in the 700–900 nm spectrum into the human cortex, and detecting the unabsorbed reflected light based on the sensitivity of blood oxygen to the wavelength and the light attenuation of the wavelength; thus elevated oxygenated hemoglobin concentrations can be used as a proxy for brain activation. In the field of cognitive neuroscience, the application of fNIRS has grown rapidly in recent decades, mainly due to its advantages over other neuroimaging modalities (fMRI, EEG) in terms of non-invasiveness, convenience, high spatial resolution, high temporal resolution, insensitivity to body movements and flexible application to a variety of experimental scenarios (inside and outside the laboratory) (Pinti et al., 2020). fNIRS is now being used not only in the fields of medicine and psychology but also in the humanities, social sciences, engineering, and other fields.

**FIGURE 12:**
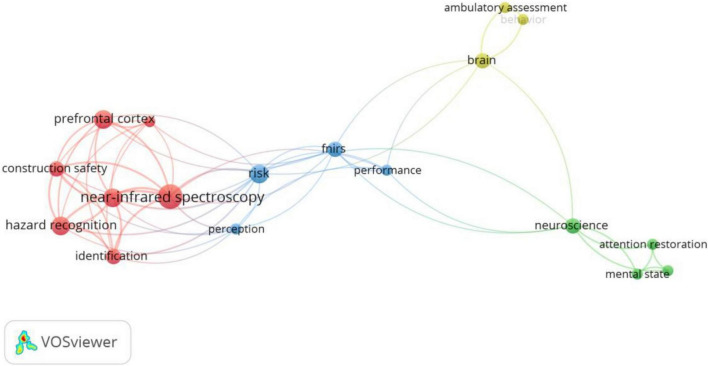
Keywords co-occurrence network of fNIRs.

Figure 12 shows the co-word network of fNIRs used in building construction research. This tool is not extensively used at the moment, and it is mainly used for the research on hazard identification in construction safety. Hazard recognition is a visual search and cognitive processing process, fNIRS recordings of workers’ prefrontal cortex (PFC) activity reveal a negative association between PFC activity and hazard recognition ability (Zhang et al., 2021). During hazard recognition, different cortical regions of the PFC are differentially and continuously activated, with the left PFC being more involved, the dorsolateral PFC is used for electrical and shock-related hazard recognition, and the ventral PFC being used for stab-related hazard recognition (Zhou et al., 2021). However, when compared to other hazards such as electrical and fire hazards, fall hazards activate the brain more and use significant cognitive resources (Liao et al., 2021). Additionally, some researchers have also developed an HRA index to measure workers’ ability to identify hazards using PFC activation data obtained *via* fNIRS (Sun and Liao, 2019). Meanwhile, fNIRs are being employed in indoor wayfinding research, where they are used to assist in the design of adaptive wayfinding systems by monitoring and classifying real-time cognitive load (Zhu et al., 2021).

## Conclusion

Over the last two decades, neuroscience tools, have expanded beyond medical and psychological applications to include humanities, social sciences, engineering, and other disciplines, resulting in the cross-fertilization of interdisciplinary knowledge with human-related research fields in these disciplines. The discipline of building construction is a perfect example of the interdisciplinary application of neuroscience tools. Given that the construction industry involves human physiological and psychological states, both for practitioners and building users, the application of neuroscience tools can significantly aid research in the field of building construction, ultimately achieving the overall goal of improving building safety, comfort, and health. Thus, this paper uses bibliometric approaches to examine the general knowledge overview and the specific knowledge structure and knowledge topics of neuroscience tools used in the field of building construction to determine how these tools advance scientific research in the field of building. In general, the main conclusions are as follows:

Knowledge overview: In the last decade, neuroscience tools have gained increasing attention from scholars in the field of building construction, and the number of related research literature has grown exponentially. ET and EEG are the most frequently used tools, primarily because ET can discover workers’ and indoor personnel’s visual search patterns, and EEG can monitor the mental power and cognitive status of workers and indoor personnel in real-time. Both of these tools may be mounted on wearable sensing devices, making them more suitable for non-laboratory research in the field of building construction. China and the United States currently have the strongest research capacities in this interdisciplinary field. Simultaneously, the majority of scientific research institutions and scholars who have made significant contributions to this field are from these two countries, indicating that these institutions and scholars are setting the future development trend for this interdisciplinary field. Additionally, the overwhelming majority of literature in this interdisciplinary field is published in journals with an impact factor of 2.5 and 8, demonstrating that the use of neuroscience tools to study building construction issues has been recognized by the mainstream academic community.

Knowledge structure: Using co-word analysis methods, the knowledge structure division of research on the application of neuroscience tools in the building construction field reveals that the research is mainly clustered into four directions, namely, built environment, construction safety, architectural design, and occupational health. In terms of specific neuroscience tools, ECG is mainly used to study the built environment; EMG is mainly used to study occupational health; fMRI is mainly used to study architectural design, and TNS currently includes only one article on the study of construction safety. EDA, ET, EEG, and fNIRs are used in the first three research directions, with ET and EEG being the most commonly used in the field of building construction and involved in research on topics such as thermal environment, acoustic environment, light environment, and air quality in the built environment. There is also research on topics such as hazard identification, mental fatigue, safety training, attention and alertness, stress levels in construction safety, aesthetic design, spatial design, and biophilic design in architectural design.

This paper attempts to present an exhaustive and systematic assessment of the application of neuroscience tools in the field of building construction, but there are some limitations. First and foremost, this research focuses on the application of neuroscience tools in the construction of buildings. The research literature on civil engineering applications such as bridges, roads, and subways is excluded throughout the process of data screening. In the future, we will be able to thoroughly investigate the application of neuroscience tools in the field of civil engineering. Secondly, only co-word analysis is used in this study to investigate the application of neuroscience tools, but co-citation analysis and main path analysis can be used in the future to uncover the highly cited literature and the citation relationships between the literature in this study. Although there are some limitations, preliminary research in this paper suggests that neuroscience tools used for construction problems are an emerging frontier research direction that provides a powerful methodological tool for human-related research problems across the construction projects’ life cycle.

## Author Contributions

MW designed the study and wrote the manuscript. XL wrote the manuscript. YL, WC, and ZW analyzed the data. XG revised the manuscript. All authors contributed to the article and approved the submitted version.

## Conflict of Interest

The authors declare that the research was conducted in the absence of any commercial or financial relationships that could be construed as a potential conflict of interest.

## Publisher’s Note

All claims expressed in this article are solely those of the authors and do not necessarily represent those of their affiliated organizations, or those of the publisher, the editors and the reviewers. Any product that may be evaluated in this article, or claim that may be made by its manufacturer, is not guaranteed or endorsed by the publisher.
