# Supplementary material for: Application of Neuroscience Tools in Building Construction – An Interdisciplinary Analysis
Source: Front Neurosci. 2022 Jun 21;16:895666. doi: 10.3389/fnins.2022.895666 (PMC9253515; doi:10.3389/fnins.2022.895666)
Supplement: Supplementary file 1 [file Data_Sheet_1.docx]

**Appendix A**

Table A1 Specific search information for each database

| **Search string** | **Database** | **Search items** | **Selection criteria** | **Initial results** |
| --- | --- | --- | --- | --- |
| electrocardiogram OR electromyogra* OR “electrodermal activity” OR “galvanic skin response” OR “skin conductance responses” OR “skin conductance level” OR “eye track*” OR electroencephalogra* OR electro-encephalography OR “event related potential*" OR “functional near-infrared spectroscopy” OR “functional magnetic resonance imaging” OR “trigeminal nerve stimulation” | Web of Science | 10041 | Noun meaning of building | 312 |
|  | Scopus | 15233 |  | 323 |
|  | PubMed | 4410 |  | 43 |

**Appendix B**

Table B1 Specific information of 307 literatures from WoS, Scopus, PubMed

| **Serial Number** | **Article Title** | **Source Title** | **Publication Year** | **DOI** |
| --- | --- | --- | --- | --- |
| 1 | A critical review on the impact of built environment on users' measured brain activity | Architectural Science Review | 2021 | 10.1080/00038628.2020.1749980 |
| 2 | A Difference in Perspective: Impact of Different Formats of Engineering Information and Spatial Cognition on Craft-Worker Eye-Gaze Patterns | Journal of Construction Engineering and Management | 2019 | 10.1061/(ASCE)CO.1943-7862.0001706 |
| 3 | A framework for evaluating muscle activity during repetitive manual material handling in construction manufacturing | Automation in Construction | 2017 | 10.1016/j.autcon.2017.01.005 |
| 4 | A multicomponent and neurophysiological intervention for the emotional and mental states of high-altitude construction workers | Automation in Construction | 2019 | 10.1016/j.autcon.2019.102836 |
| 5 | A multimodal study to measure the cognitive demands of hazard recognition in construction workplaces | Safety Science | 2021 | 10.1016/j.ssci.2020.105010 |
| 6 | A neuro-cognitive investigation of the impact of glass floors on people | Architectural Science Review | 2021 | 10.1080/00038628.2020.1858574 |
| 7 | A neurocognitive study of the emotional impact of geometrical criteria of architectural space | Architectural Science Review | 2021 | 10.1080/00038628.2021.1940827 |
| 8 | A psychophysiological effect of indoor thermal condition on college students' learning performance through EEG measurement | Building and Environment | 2020 | 10.1016/j.buildenv.2020.107223 |
| 9 | A quantitative study for indoor workplace biophilic design to improve health and productivity performance | Journal of Cleaner Production | 2021 | 10.1016/j.jclepro.2021.129168 |
| 10 | A study of preferred colour rendering of light sources: Shop lighting | Lighting Research & Technology | 2016 | 10.1177/1477153515573042 |
| 11 | A Study on the Comparison of the Visual Attention Characteristics on the Facade Image of a Detached House Due to the Features on Windows | Journal of Asian Architecture and Building Engineering | 2016 | 10.3130/jaabe.15.209 |
| 12 | A Systematic Review of Eye-Tracking Studies of Construction Safety | Frontiers in Neuroscience | 2022 | 10.3389/fnins.2022.891725 |
| 13 | A systematic review of the attributes of interior design affecting user’s positive emotions measured via bio-signals | Journal of The Architectural institute of Korea | 2020 | 10.5659/JAIK_PD.2020.36.5.83 |
| 14 | Affective response to architecture - investigating human reaction to spaces with different geometry | Architectural Science Review | 2017 | 10.1080/00038628.2016.1266597 |
| 15 | Ambient Particulate Air Pollution and EctopyThe Environmental Epidemiology of Arrhythmogenesis in Women's Health Initiative Study, 1999-2004 | Journal of Toxicology and Environmental Health-Part A-Current Issues | 2009 | 10.1080/15287390802445483 |
| 16 | An analysis of the characteristics of the observer's perception of the elevation image-according to the change in the shape of the house- | Journal of The Architectural institute of Korea | 2020 | 10.5659/JAIK.2020.36.12.89 |
| 17 | An automatic and non-invasive physical fatigue assessment method for construction workers | Automation in Construction | 2019 | 10.1016/j.autcon.2019.02.020 |
| 18 | An EEG Analysis of the Effects of Color and Ceiling Height on the Perceived Restorativeness of Users-Focused on Indoor Common Spaces of Educational Facilities | Journal of The Architectural institute of Korea | 2022 | 10.5659/JAIK.2022.38.2.63 |
| 19 | An EMG-based ergonomic evaluation of manual bar bending | international Journal of industrial and Systems Engineering | 2007 | 10.1504/IJISE.2007.012464 |
| 20 | An EMG-based model to estimate lumbar muscle forces and spinal loads during complex, high-effort tasks: Development and application to residential construction using prefabricated walls | international Journal of industrial Ergonomics | 2011 | 10.1016/j.ergon.2011.03.004 |
| 21 | An instance-based scoring system for indoor landmark salience evaluation | Geografie | 2019 | 10.37040/geografie2019124020103 |
| 22 | An Investigation of the Effects of Changes in the Indoor Ambient Temperature on Arousal Level, Thermal Comfort, and Physiological Indices | Applied Sciences-Basel | 2019 | 10.3390/app9050899 |
| 23 | Analysis of human electroencephalogram features in different indoor environments | Building and Environment | 2020 | 10.1016/j.buildenv.2020.107328 |
| 24 | Analysis of individual and occupational risk factors on task performance and biomechanical demands for a simulated drilling task | international Journal of industrial Ergonomics | 2010 | 10.1016/j.ergon.2010.06.003 |
| 25 | Applicability of eye trackers in marketing activities related to historical monuments. Comparison of experts' predictions and visual reactions of non-professionals | Journal of Cultural Heritage | 2021 | 10.1016/j.culher.2021.02.004 |
| 26 | Application of Data Fusion via Canonical Polyadic Decomposition in Risk Assessment of Musculoskeletal Disorders in Construction: Procedure and Stability Evaluation | Journal of Construction Engineering and Management | 2021 | 10.1061/(ASCE)CO.1943-7862.0002106 |
| 27 | Application of Functional Near-Infrared Spectroscopy to Measure Engineering Decision-Making and Design Cognition: Literature Review and Synthesis of Methods | Journal of Computing in Civil Engineering | 2019 | 10.1061/(ASCE)CP.1943-5487.0000848 |
| 28 | Application of Wearable Biosensors to Construction Sites. I: Assessing Workers' Stress | Journal of Construction Engineering and Management | 2019 | 10.1061/(ASCE)CO.1943-7862.0001729 |
| 29 | Application of Wearable Biosensors to Construction Sites. II: Assessing Workers' Physical Demand | Journal of Construction Engineering and Management | 2019 | 10.1061/(ASCE)CO.1943-7862.0001710 |
| 30 | Application of wearable EEG sensors for indoor thermal comfort measurements | Acta Imeko | 2021 | 10.21014/acta_imeko.v10i4.1180 |
| 31 | Applications of electroencephalography in construction | Automation in Construction | 2022 | 10.1016/j.autcon.2021.103985 |
| 32 | Architectural design and the brain: Effects of ceiling height and perceived enclosure on beauty judgments and approach-avoidance decisions | Journal of Environmental Psychology | 2015 | 10.1016/j.jenvp.2014.11.006 |
| 33 | Are Visual Search Patterns Predictive of Hazard Recognition Performance? Empirical Investigation Using Eye-Tracking Technology | Journal of Construction Engineering and Management | 2019 | 10.1061/(ASCE)CO.1943-7862.0001589 |
| 34 | Assessing occupational risk of heat stress at construction: A worker-centric wearable sensor-based approach | Safety Science | 2021 | 10.1016/j.ssci.2021.105395 |
| 35 | Assessing Task Mental Workload in Construction Projects: A Novel Electroencephalography Approach | Journal of Construction Engineering and Management | 2017 | 10.1061/(ASCE)CO.1943-7862.0001345 |
| 36 | Assessing Work-Related Risk Factors on Low Back Disorders among Roofing Workers | Journal of Construction Engineering and Management | 2017 | 10.1061/(ASCE)CO.1943-7862.0001320 |
| 37 | Assessment of a passive exoskeleton system on spinal biomechanics and subjective responses during manual repetitive handling tasks among construction workers | Safety Science | 2021 | 10.1016/j.ssci.2021.105382 |
| 38 | Assessment of construction workers' perceived risk using physiological data from wearable sensors: A machine learning approach | Journal of Building Engineering | 2021 | 10.1016/j.jobe.2021.102824 |
| 39 | Assessment of mental fatigue using electroencephalography (EEG) and virtual reality (VR) for construction fall hazard prevention | Engineering Construction and Architectural Management | 2021 | 10.1108/ECAM-01-2021-0017 |
| 40 | Assessment of Personal Relaxation in Indoor-Air Environments: Study in Real Full-Scale Laboratory Houses | international Journal of Environmental Research and Public Health | 2021 | 10.3390/ijerph181910246 |
| 41 | Automated classification of indoor environmental quality control using stacked ensembles based on electroencephalograms | Computer-Aided Civil and infrastructure Engineering | 2020 | 10.1111/mice.12515 |
| 42 | Automating and scaling personalized safety training using eye-tracking data | Automation in Construction | 2018 | 10.1016/j.autcon.2018.05.006 |
| 43 | Autonomic Nervous System Responses to Viewing Green and Built Settings: Differentiating Between Sympathetic and Parasympathetic Activity | international Journal of Environmental Research and Public Health | 2015 | 10.3390/ijerph121215026 |
| 44 | Biomechanical analysis of risk factors for work-related musculoskeletal disorders during repetitive lifting task in construction workers | Automation in Construction | 2017 | 10.1016/j.autcon.2017.07.007 |
| 45 | Blue light effect on EEG activity - The role of exposure timing and chronotype | Lighting Research & Technology | 2020 | 10.1177/1477153519876969 |
| 46 | Brain Electrical Responses to High- and Low-Ranking Buildings | Clinical Eeg and Neuroscience | 2009 | 10.1177/155005940904000307 |
| 47 | Brain functional connectivity differs when viewing pictures from natural and built environments using fMRI resting state analysis | Scientific Reports | 2021 | 10.1038/s41598-021-83246-5 |
| 48 | Brain-computer interface for hands-free teleoperation of construction robots | Automation in Construction | 2021 | 10.1016/j.autcon.2020.103523 |
| 49 | Brainwave-driven human-robot collaboration in construction | Automation in Construction | 2021 | 10.1016/j.autcon.2021.103556 |
| 50 | Building environment information and human perceptual feedback collected through a combined virtual reality (VR) and electroencephalogram (EEG) method | Energy and Buildings | 2020 | 10.1016/j.enbuild.2020.110259 |
| 51 | Building Vulnerability in a Changing Climate: Indoor Temperature Exposures and Health Outcomes in Older Adults Living in Public Housing during an Extreme Heat Event in Cambridge, MA | international Journal of Environmental Research and Public Health | 2019 | 10.3390/ijerph16132373 |
| 52 | Can a metric combining arm elevation and trapezius muscle activity predict neck/shoulder pain? A prospective cohort study in construction and healthcare | international Archives of Occupational and Environmental Health | 2021 | 10.1007/s00420-020-01610-w |
| 53 | Cardiorespiratory and Thermoregulatory Parameters Are Good Surrogates for Measuring Physical Fatigue during a Simulated Construction Task | international Journal of Environmental Research and Public Health | 2020 | 10.3390/ijerph17155418 |
| 54 | Cardiorespiratory responses to low-level ozone exposure: The inDoor Ozone Study in childrEn (DOSE) | Environment international | 2019 | 10.1016/j.envint.2019.105021 |
| 55 | Changes in EEG signals during the cognitive activity at varying air temperature and relative humidity | Journal of Exposure Science and Environmental Epidemiology | 2020 | 10.1038/s41370-019-0154-1 |
| 56 | Choice zones: architecturally relevant areas of interest | Spatial Cognition and Computation | 2018 | 10.1080/13875868.2017.1412443 |
| 57 | Circadian-effective light and its impact on alertness in office workers | Lighting Research & Technology | 2019 | 10.1177/1477153517750006 |
| 58 | Classification of construction hazard-related perceptions using: Wearable electroencephalogram and virtual reality | Automation in Construction | 2021 | 10.1016/j.autcon.2021.103975 |
| 59 | Cognitive response and how it is affected by changes in temperature | Building Research and information | 2021 | 10.1080/09613218.2020.1800439 |
| 60 | Cold and warm coloured classrooms. Effects on students' attention and memory measured through psychological and neurophysiological responses | Building and Environment | 2021 | 10.1016/j.buildenv.2021.107726 |
| 61 | Combined effects of noise and air temperature on human neurophysiological responses in a simulated indoor environment | Applied Ergonomics | 2020 | 10.1016/j.apergo.2020.103189 |
| 62 | Comfortable Indoor Lighting Conditions Evaluated from Psychological and Physiological Responses | Leukos | 2016 | 10.1080/15502724.2015.1061945 |
| 63 | Comfortable indoor lighting conditions for LEDlights evaluated from psychological and physiological responses | Applied Ergonomics | 2020 | 10.1016/j.apergo.2019.102941 |
| 64 | Comfortably cool bedroom environment during the initial phase of the sleeping period delays the onset of sleep in summer | Building and Environment | 2016 | 10.1016/j.buildenv.2016.03.030 |
| 65 | Comparing the roles of landmark visual salience and semantic salience in visual guidance during indoor wayfinding | Cartography and Geographic information Science | 2020 | 10.1080/15230406.2019.1697965 |
| 66 | Comparing written and photo-based indoor wayfinding instructions through eye fixation measures and user ratings as mental effort assessments | Journal of Eye Movement Research | 2019 | 10.16910/jemr.12.1.1 |
| 67 | Consumer Response to Novel Indoor Foliage Plant Attributes: Evidence from a Conjoint Experiment and Gaze Analysis | Hortscience | 2015 | 10.21273/hortsci.50.10.1524 |
| 68 | Cortical activation to indoor versus outdoor scenes: an fMRI study | Experimental Brain Research | 2007 | 10.1007/s00221-006-0766-2 |
| 69 | Cross-cultural differences in processing of architectural ranking: Evidence from an event-related potential study | Cognitive Neuroscience | 2014 | 10.1080/17588928.2013.869740 |
| 70 | Data Quality and Reliability Assessment of Wearable EMG and IMU Sensor for Construction Activity Recognition | Sensors | 2020 | 10.3390/s20185264 |
| 71 | Day-to-day particulate exposures and health changes in Los Angeles area residents with severe lung disease | Journal of The Air & Waste Management Association | 1999 | 10.1080/10473289.1999.10463890 |
| 72 | Decreasing the physical workload of construction work with the use of four auxiliary handling devices | international Journal of industrial Ergonomics | 1999 | 10.1016/S0169-8141(98)00030-4 |
| 73 | Degree of eye opening: A new discomfort glare indicator | Building and Environment | 2015 | 10.1016/j.buildenv.2014.11.010 |
| 74 | Design and construction strategies for reducing embodied impacts from buildings - Case study analysis | Energy and Buildings | 2018 | 10.1016/j.enbuild.2018.01.033 |
| 75 | Detecting and measuring construction workers' vigilance through hybrid kinematic-EEG signals | Automation in Construction | 2019 | 10.1016/j.autcon.2018.12.018 |
| 76 | Determining how different levels of indoor carbon dioxide affect human monotonous task performance and their effects on human activation states using a lab experiment: a tracking task | Ergonomics | 2020 | 10.1080/00140139.2020.1784466 |
| 77 | Development and Testing of a Personalized Hazard-Recognition Training Intervention | Journal of Construction Engineering and Management | 2017 | 10.1061/(ASCE)CO.1943-7862.0001256 |
| 78 | Differences between EEG during thermal discomfort and thermal displeasure | Building and Environment | 2021 | 10.1016/j.buildenv.2021.108220 |
| 79 | Distinct Familiarity-Based Response Patterns for Faces and Buildings in Perirhinal and Parahippocampal Cortex | Journal of Neuroscience | 2013 | 10.1523/JNEUROSCI.0126-13.2013 |
| 80 | Distributed subordinate specificity for bodies, faces, and buildings in human ventral visual cortex | Neuroimage | 2010 | 10.1016/j.neuroimage.2009.11.022 |
| 81 | Do plants affect brainwaves? Effect of indoor plants in work environment on mental stress | European Journal of Horticultural Science | 2020 | 10.17660/eJHS.2020/85.4.9 |
| 82 | Does Augmented Reality Effectively Foster Visual Learning Process in Construction? An Eye-Tracking Study in Steel Installation | Advances in Civil Engineering | 2018 | 10.1155/2018/2472167 |
| 83 | EEG Signal-Processing Framework to Obtain High-Quality Brain Waves from an Off-the-Shelf Wearable EEG Device | Journal of Computing in Civil Engineering | 2018 | 10.1061/(ASCE)CP.1943-5487.0000719 |
| 84 | EEG-based work experience prediction using hazard recognition | Automation in Construction | 2022 | 10.1016/j.autcon.2022.104151 |
| 85 | EEG-based workers' stress recognition at construction sites | Automation in Construction | 2018 | 10.1016/j.autcon.2018.05.027 |
| 86 | Effect of a personal weight transfer device on muscle activities and joint flexions in the stooped posture | Journal of Electromyography and Kinesiology | 2013 | 10.1016/j.jelekin.2012.08.014 |
| 87 | Effect of concrete block weight and wall height on electromyographic activity and heart rate of masons | Ergonomics | 2005 | 10.1080/00140130500274168 |
| 88 | Effect of daylight LED on visual comfort, melatonin, mood, waking performance and sleep | Lighting Research & Technology | 2019 | 10.1177/1477153519828419 |
| 89 | Effect of Fragrant Primula Flowers on Physiology and Psychology in Female College Students: An Empirical Study | Frontiers in Psychology | 2021 | 10.3389/fpsyg.2021.607876 |
| 90 | Effect of Indoors Artificial Lighting Conditions on Computer-Based Learning Performance | international Journal of Environmental Research and Public Health | 2020 | 10.3390/ijerph17072537 |
| 91 | Effect of intensity of short-wavelength light on electroencephalogram and subjective alertness | Lighting Research & Technology | 2020 | 10.1177/1477153519872801 |
| 92 | Effect of long-wavelength light on electroencephalogram and subjective alertness | Lighting Research & Technology | 2020 | 10.1177/1477153520902255 |
| 93 | Effect of Road and Railway Sound on Psychological and Physiological Responses in an Office Environment | Buildings | 2022 | 10.3390/buildings12010006 |
| 94 | Effect of sound environment on learning efficiency in classrooms | Journal of Environmental Engineering | 2010 | 10.3130/aije.75.561 |
| 95 | Effect of temperature on attention ability based on electroencephalogram measurements | Building and Environment | 2019 | 10.1016/j.buildenv.2018.10.020 |
| 96 | Effect of thermal sensation on emotional responses as measured through brain waves | Building and Environment | 2017 | 10.1016/j.buildenv.2017.03.023 |
| 97 | Effects of a cool classroom microclimate on cardiac autonomic control and cognitive performances in undergraduate students | Science of The Total Environment | 2022 | 10.1016/j.scitotenv.2021.152005 |
| 98 | Effects of a Participatory Ergonomics Intervention With Wearable Technical Measurements of Physical Workload in the Construction Industry: Cluster Randomized Controlled Trial | Journal of Medical internet Research | 2018 | 10.2196/10272 |
| 99 | Effects of biophilic indoor environment on stress and anxiety recovery: A between-subjects experiment in virtual reality | Environment international | 2020 | 10.1016/j.envint.2019.105427 |
| 100 | Effects of biophilic interventions in office on stress reaction and cognitive function: A randomized crossover study in virtual reality | indoor Air | 2019 | 10.1111/ina.12593 |
| 101 | Effects of Changes to Architectural Elements on Human Relaxation-Arousal Responses: Based on VR and EEG | international Journal of Environmental Research and Public Health | 2021 | 10.3390/ijerph18084305 |
| 102 | Effects of different ambient environments on human responses and work performance | Journal of Ambient intelligence and Humanized Computing | 2016 | 10.1007/s12652-016-0393-0 |
| 103 | Effects of fine particulate on heart rate variability in Beijing: a panel study of healthy elderly subjects | international Archives of Occupational and Environmental Health | 2012 | 10.1007/s00420-011-0646-3 |
| 104 | Effects of floor impact noise on psychophysiological responses | Building and Environment | 2017 | 10.1016/j.buildenv.2017.02.005 |
| 105 | Effects of indoor foliage plants on college students' work attention recovery: An experiment focusing on plant characteristics | European Journal of Horticultural Science | 2021 | 10.17660/eJHS.2021/86.6.8 |
| 106 | Effects of load carrying techniques on gait parameters, dynamic balance, and physiological parameters during a manual material handling task | Engineering Construction and Architectural Management | 2021 | 10.1108/ECAM-03-2021-0245 |
| 107 | Effects of noise sensitivity on psychophysiological responses to building noise | Building and Environment | 2018 | 10.1016/j.buildenv.2018.03.061 |
| 108 | Effects of physical fatigue on the induction of mental fatigue of construction workers: A pilot study based on a neurophysiological approach | Automation in Construction | 2020 | 10.1016/j.autcon.2020.103381 |
| 109 | Effects of plantscape colors on psycho-physiological responses of university students | Journal of Food Agriculture & Environment | 2012 | Null |
| 110 | Effects of pre-sleep thermal environment on human thermal state and sleep quality | Building and Environment | 2019 | 10.1016/j.buildenv.2018.11.035 |
| 111 | Effects of viewing flowering plants on employees' wellbeing in an office-like environment | indoor and Built Environment | 2021 | 10.1177/1420326X20942572 |
| 112 | Effects of working posture and roof slope on activation of lower limb muscles during shingle installation | Ergonomics | 2020 | 10.1080/00140139.2020.1772378 |
| 113 | E-happiness physiological indicators of construction workers' productivity: A machine learning approach | Journal of Asian Architecture and Building Engineering | 2019 | 10.1080/13467581.2019.1687090 |
| 114 | Electrocardiogram abnormalities in residents in cold homes: a cross-sectional analysis of the nationwide Smart Wellness Housing survey in Japan | Environmental Health and Preventive Medicine | 2021 | 10.1186/s12199-021-01024-1 |
| 115 | Electroencephalographic Correlates of Sensorimotor Integration and Embodiment during the Appreciation of Virtual Architectural Environments | Frontiers in Psychology | 2015 | 10.3389/fpsyg.2015.01944 |
| 116 | Electromyographic evaluation of different handle shapes of masons' trowels | international Journal of Occupational Safety and Ergonomics | 2021 | 10.1080/10803548.2018.1530489 |
| 117 | Electromyographic measures of muscle activation and changes in muscle architecture of human elbow flexors during fatiguing contractions | Journal of Applied Physiology | 2008 | 10.1152/japplphysiol.01058.2007 |
| 118 | Elevated airflow can maintain sleep quality and thermal comfort of the elderly in a hot environment | indoor Air | 2019 | 10.1111/ina.12599 |
| 119 | Emotion and novelty processing in an implicit aesthetic experience of architectures: evidence from an event-related potential study | Neuroreport | 2015 | 10.1097/WNR.0000000000000344 |
| 120 | Emotions and physiological responses elicited by neighbours sounds in wooden residential buildings | Building and Environment | 2022 | 10.1016/j.buildenv.2021.108729 |
| 121 | Encoding Pleasant and Unpleasant Expression of the Architectural Window Shapes: An ERP Study | Frontiers in Behavioral Neuroscience | 2019 | 10.3389/fnbeh.2019.00186 |
| 122 | Environmental Distress and Physiological Signals: Examination of the Saliency Detection Method | Journal of Computing in Civil Engineering | 2020 | 10.1061/(ASCE)CP.1943-5487.0000926 |
| 123 | Ergonomic evaluation of a fixture used for power driven wire-tying hand tools | international Journal of industrial Ergonomics | 2003 | 10.1016/S0169-8141(03)00030-1 |
| 124 | Evaluating indoor pedestrian navigation interfaces using mobile eye tracking | Spatial Cognition and Computation | 2017 | 10.1080/13875868.2016.1219913 |
| 125 | Evaluating the attitudes of different trainee groups towards eye tracking enhanced safety training methods | Advanced Engineering informatics | 2021 | 10.1016/j.aei.2021.101353 |
| 126 | Evaluating the effectiveness of biometric sensors and their signal features for classifying human experience in virtual environments | Advanced Engineering informatics | 2021 | 10.1016/j.aei.2021.101358 |
| 127 | Evaluating the impact of mental fatigue on construction equipment operators' ability to detect hazards using wearable eye-tracking technology | Automation in Construction | 2019 | 10.1016/j.autcon.2019.102835 |
| 128 | Evaluation of calculation methods of mean skin temperature for use in thermal comfort study | Building and Environment | 2011 | 10.1016/j.buildenv.2010.08.011 |
| 129 | Evaluation of indoor guidance systems using eye tracking in an immersive virtual environment | Spatial Cognition and Computation | 2017 | 10.1080/13875868.2016.1228654 |
| 130 | Evaluation of Wood Coverage on Building Facades Towards Sustainability | Sustainability | 2019 | 10.3390/su11051407 |
| 131 | Evaluation on the performance of quilts based on young people's sleep quality and thermal comfort in winter | Energy and Buildings | 2019 | 10.1016/j.enbuild.2018.10.040 |
| 132 | Examining the Relationship between Construction Workers' Visual Attention and Situation Awareness under Fall and Tripping Hazard Conditions: Using Mobile Eye Tracking | Journal of Construction Engineering and Management | 2018 | 10.1061/(ASCE)CO.1943-7862.0001516 |
| 133 | Examining the validity of the total dwell time of eye fixations to identify landmarks in a building | Journal of Eye Movement Research | 2016 | 10.16910/jemr.9.3.4 |
| 134 | Examining wayfinding behaviours in architectural spaces using brain imaging with electroencephalography (EEG) | Architectural Science Review | 2018 | 10.1080/00038628.2018.1523129 |
| 135 | Experimental Evaluation of the Indoor Human Thermal Comfort under Different Environments Based on Heart Rate Variability Analysis | Environmental Science and Engineering | 2020 | 10.1007/978-981-13-9520-8_15 |
| 136 | Experimental study on thermal comfort of sleeping people at different air temperatures | Building and Environment | 2014 | 10.1016/j.buildenv.2013.11.024 |
| 137 | Exploring a sustainable building's impact on occupant mental health and cognitive function in a virtual environment | Scientific Reports | 2021 | 10.1038/s41598-021-85210-9 |
| 138 | Exploring construction workers' brain connectivity during hazard recognition: a cognitive psychology perspective | international Journal of Occupational Safety and Ergonomics | 2022 | 10.1080/10803548.2022.2035966 |
| 139 | Exploring eye-tracking searching strategies for construction hazard recognition in a laboratory scene | Safety Science | 2019 | 10.1016/j.ssci.2019.08.012 |
| 140 | Exploring inattentional blindness in failure of safety risk perception: Focusing on safety knowledge in construction industry | Safety Science | 2022 | 10.1016/j.ssci.2021.105518 |
| 141 | Exploring the physiological, neurophysiological and cognitive performance effects of elevated carbon dioxide concentrations indoors | Building and Environment | 2019 | 10.1016/j.buildenv.2019.04.010 |
| 142 | Exploring the relations between the physiological factors and the likelihood of accidents on construction sites | Engineering Construction and Architectural Management | 2020 | 10.1108/ECAM-11-2020-0958 |
| 143 | Externally-induced meditative states: an exploratory fMRI study of architects' responses to contemplative architecture | Frontiers of Architectural Research | 2017 | 10.1016/j.foar.2017.02.002 |
| 144 | Eye Tracking to Evaluate the Usability of an Online Pneumoconiosis Education Booklet in a Sample of South Asian Construction Workers | Cin-Computers informatics Nursing | 2020 | 10.1097/CIN.0000000000000635 |
| 145 | Eye-Tracking Experimental Study Investigating the Influence Factors of Construction Safety Hazard Recognition | Journal of Construction Engineering and Management | 2020 | 10.1061/(ASCE)CO.1943-7862.0001884 |
| 146 | Eye-tracking the city: Matching the design of streetscapes in high-rise environments with users’ visual experiences | Journal of Digital Landscape Architecture | 2020 | 10.14627/537690038 |
| 147 | Feasibility analysis of electrodermal activity (EDA) acquired from wearable sensors to assess construction workers' perceived risk | Safety Science | 2019 | 10.1016/j.ssci.2019.01.022 |
| 148 | Feasibility Study to Identify Brain Activity and Eye-Tracking Features for Assessing Hazard Recognition Using Consumer-Grade Wearables in an Immersive Virtual Environment | Journal of Construction Engineering and Management | 2021 | 10.1061/(ASCE)CO.1943-7862.0002130 |
| 149 | Follow people or signs? A novel way-finding method based on experiments and simulation | Physica A-Statistical Mechanics and Its Applications | 2021 | 10.1016/j.physa.2021.125926 |
| 150 | Follow the evacuation signs or surrounding people during building evacuation, an experimental study | Physica A-Statistical Mechanics and Its Applications | 2020 | 10.1016/j.physa.2020.125156 |
| 151 | Fractal Dimension Calculation and Visual Attention Simulation: Assessing the Visual Character of an Architectural Facade | Buildings | 2021 | 10.3390/buildings11040163 |
| 152 | Fusing imperfect experimental data for risk assessment of musculoskeletal disorders in construction using canonical polyadic decomposition | Automation in Construction | 2020 | 10.1016/j.autcon.2020.103322 |
| 153 | Gaze and discomfort glare, Part 1: Development of a gaze-driven photometry | Lighting Research & Technology | 2017 | 10.1177/1477153516649016 |
| 154 | Gaze Point in the Evacuation Drills: Analysis of Eye Movement at the Indoor Wayfinding | Sustainability | 2020 | 10.3390/su12072902 |
| 155 | Gender and Age Differences in Using Indoor Maps for Wayfinding in Real Environments | Isprs international Journal of Geo-information | 2019 | 10.3390/ijgi8010011 |
| 156 | Glare indicators: an analysis of ocular behaviour in an office equipped with venetian blinds | indoor and Built Environment | 2016 | 10.1177/1420326X14538082 |
| 157 | Hazard differentiation embedded in the brain: A near-infrared spectroscopy-based study | Automation in Construction | 2021 | 10.1016/j.autcon.2020.103473 |
| 158 | Health effects of particulate air pollution: A review of epidemiological evidence | inhalation Toxicology | 2011 | 10.3109/08958378.2011.593587 |
| 159 | Heart rate variability at different thermal comfort levels | European Journal of Applied Physiology | 2008 | 10.1007/s00421-008-0718-6 |
| 160 | Heart rate variation and electroencephalograph - the potential physiological factors for thermal comfort study | indoor Air | 2009 | 10.1111/j.1600-0668.2008.00565.x |
| 161 | Higher light intensity induces modulations in brain activity even during regular daytime working hours | Lighting Research & Technology | 2016 | 10.1177/1477153515576399 |
| 162 | How does gender affect indoor wayfinding under time pressure? | Cartography and Geographic information Science | 2020 | 10.1080/15230406.2020.1760940 |
| 163 | Human cognitive functions and psycho-physiological responses under low thermal conditions in a simulated office environment | Work-A Journal of Prevention Assessment & Rehabilitation | 2021 | 10.3233/WOR-213469 |
| 164 | Human exposure to aerosol from indoor gas stove cooking and the resulting nervous system responses | indoor Air | 2022 | 10.1111/ina.12983 |
| 165 | Human physiological responses to wooden indoor environment | Physiology & Behavior | 2017 | 10.1016/j.physbeh.2017.02.043 |
| 166 | Human response to window views and indoor plants in the workplace | Hortscience | 2005 | 10.21273/hortsci.40.5.1354 |
| 167 | Human-building interaction under various indoor temperatures through neural-signal electroencephalogram (EEG) methods | Building and Environment | 2018 | 10.1016/j.buildenv.2017.12.004 |
| 168 | Hybrid deep-learning model to recognise emotional responses of users towards architectural design alternatives | Journal of Asian Architecture and Building Engineering | 2019 | 10.1080/13467581.2019.1660663 |
| 169 | Identification and classification of construction equipment operators' mental fatigue using wearable eye-tracking technology | Automation in Construction | 2020 | 10.1016/j.autcon.2019.103000 |
| 170 | Identification of Biomechanical Risk Factors for the Development of Lower-Back Disorders during Manual Rebar Tying | Journal of Construction Engineering and Management | 2017 | 10.1061/(ASCE)CO.1943-7862.0001208 |
| 171 | Impact of Construction Workers' Hazard Identification Skills on Their Visual Attention | Journal of Construction Engineering and Management | 2017 | 10.1061/(ASCE)CO.1943-7862.0001373 |
| 172 | Impact of contour on aesthetic judgments and approach-avoidance decisions in architecture | Proceedings of The National Academy of Sciences of The United States of America | 2013 | 10.1073/pnas.1301227110 |
| 173 | Impact of indoor thermal comfort on physiological parameters of human body | Journal of Central South University of Technology | 2009 | 10.1007/s11771-009-0176-2 |
| 174 | Implications of neuroarchitecture for the experience of the built environment: a scoping review | Archnet-Ijar international Journal of Architectural Research | 2021 | 10.1108/ARCH-09-2021-0249 |
| 175 | Indoor nature exposure and influence on physiological stress markers | international Journal of Environmental Health Research | 2021 | 10.1080/09603123.2019.1679357 |
| 176 | Influence mechanism of construction workers' safety psychology on their safety behavior based on event-related potentials | Neuroquantology | 2018 | 10.14704/nq.2018.16.6.1601 |
| 177 | Influence of knee angle and individual flexibility on the flexion-relaxation response of the low back musculature | Journal of Electromyography and Kinesiology | 2004 | 10.1016/j.jelekin.2003.12.001 |
| 178 | Influence of semantic cues on hazard-inspection performance: a case in construction safety | international Journal of Occupational Safety and Ergonomics | 2021 | 10.1080/10803548.2018.1541648 |
| 179 | Influencing factors on thermal comfort and biosignals of occupant-a review | Journal of Mechanical Science and Technology | 2021 | 10.1007/s12206-021-0832-5 |
| 180 | Integrated Approach to Evaluating the Effect of Indoor CO2 Concentration on Human Cognitive Performance and Neural Responses in Office Environment | Journal of Management in Engineering | 2022 | 10.1061/(ASCE)ME.1943-5479.0000993 |
| 181 | Investigating the effect of indoor thermal environment on occupants' mental workload and task performance using electroencephalogram | Building and Environment | 2019 | 10.1016/j.buildenv.2019.05.012 |
| 182 | Investigating the relation between electroencephalogram, thermal comfort, and cognitive performance in neutral to hot indoor environment | indoor Air | 2021 | 10.1111/ina.12941 |
| 183 | Investigation of bio-signal changes of occupants resting in buildings using local cooling and heating seats | Energy and Buildings | 2021 | 10.1016/j.enbuild.2021.111092 |
| 184 | Investigation of Gender Differences in Sleeping Comfort at Different Environmental Temperatures | indoor and Built Environment | 2012 | 10.1177/1420326X11425967 |
| 185 | Investigation of sleep quality under different temperatures based on subjective and physiological measurements | Hvac&R Research | 2012 | 10.1080/10789669.2012.667037 |
| 186 | Investigation of students? short-term memory performance and thermal sensation with heart rate variability under different environments in summer | Building and Environment | 2021 | 10.1016/j.buildenv.2021.107765 |
| 187 | Leading indicators of mental representation in construction hazard recognition | international Journal of Occupational Safety and Ergonomics | 2021 | 10.1080/10803548.2021.1952005 |
| 188 | Lighting Design for Energy Sustainability, Information, and Perception. A Museum Environment as a Case Study | Sustainability | 2018 | 10.3390/su10051671 |
| 189 | Linking the cognitive load induced by route instruction types and building configuration during indoor route guidance, a usability study in VR | international Journal of Geographical information Science | 2022 | 10.1080/13658816.2022.2032080 |
| 190 | Local body cooling to improve sleep quality and thermal comfort in a hot environment | indoor Air | 2018 | 10.1111/ina.12428 |
| 191 | Mapping landscape spaces: Methods for understanding spatial-visual characteristics in landscape design | Environmental Impact Assessment Review | 2020 | 10.1016/j.eiar.2020.106376 |
| 192 | Mapping the Evolutions and Trends of Literature on Wayfinding in Indoor Environments | European Journal of investigation in Health Psychology and Education | 2021 | 10.3390/ejihpe11020042 |
| 193 | Measurement and prediction of work engagement under different indoor lighting conditions using physiological sensing | Building and Environment | 2021 | 10.1016/j.buildenv.2021.108098 |
| 194 | Measurement of occupants' stress based on electroencephalograms (EEG) in twelve combined environments | Building and Environment | 2015 | 10.1016/j.buildenv.2014.10.003 |
| 195 | Measuring and Computing Cognitive Statuses of Construction Workers Based on Electroencephalogram: A Critical Review.. | IEEE Transactions on Computational Social Systems | 1999 | 10.1109/TCSS.2022.3158585 |
| 196 | Measuring human physiological indices for thermal comfort assessment through wearable devices: A review | Measurement | 2021 | 10.1016/j.measurement.2021.109872 |
| 197 | Measuring the Impacts of Safety Knowledge on Construction Workers' Attentional Allocation and Hazard Detection Using Remote Eye-Tracking Technology | Journal of Management in Engineering | 2017 | 10.1061/(ASCE)ME.1943-5479.0000526 |
| 198 | Measuring Workers' Emotional State during Construction Tasks Using Wearable EEG | Journal of Construction Engineering and Management | 2018 | 10.1061/(ASCE)CO.1943-7862.0001506 |
| 199 | Monitoring distraction of construction workers caused by noise using a wearable Electroencephalography (EEG) device | Automation in Construction | 2021 | 10.1016/j.autcon.2021.103598 |
| 200 | Monitoring fatigue in construction workers using physiological measurements | Automation in Construction | 2017 | 10.1016/j.autcon.2017.03.003 |
| 201 | Monitoring Physiological Reactions of Construction Workers in Virtual Environment: Feasibility Study Using Noninvasive Affective Sensors | Journal of Legal Affairs and Dispute Resolution in Engineering and Construction | 2021 | 10.1061/(ASCE)LA.1943-4170.0000480 |
| 202 | Monitoring workers' attention and vigilance in construction activities through a wireless and wearable electroencephalography system | Automation in Construction | 2017 | 10.1016/j.autcon.2017.02.001 |
| 203 | Navigation in Indoor Environments: Does the Type of Visual Learning Stimulus Matter? | Isprs international Journal of Geo-information | 2019 | 10.3390/ijgi8060251 |
| 204 | Neck Disorders among Construction Workers: Understanding the Physical Loads on the Cervical Spine during Static Lifting Tasks | industrial Health | 2010 | 10.2486/indhealth.48.145 |
| 205 | Neural representation of different 3D architectural images: An EEG study | integrated Computer-Aided Engineering | 2019 | 10.3233/ICA-180591 |
| 206 | Neural-signal electroencephalogram (EEG) methods to improve human-building interaction under different indoor air quality | Energy and Buildings | 2019 | 10.1016/j.enbuild.2019.05.055 |
| 207 | Neurobehavioral assessment of force feedback simulation in industrial robotic teleoperation | Automation in Construction | 2021 | 10.1016/j.autcon.2021.103674 |
| 208 | Neurobiological effects of urban built and natural environment on mental health: systematic review | Reviews On Environmental Health | 2021 | 10.1515/reveh-2021-0137 |
| 209 | Neurobiological relationships between ambient lighting and the startle response to acoustic stress in humans | international Journal of Neuroscience | 2001 | 10.3109/00207450108986542 |
| 210 | Neurophysiological correlates of embodiment and motivational factors during the perception of virtual architectural environments | Cognitive Processing | 2015 | 10.1007/s10339-015-0725-6 |
| 211 | Neuroscience and architecture: What does the brain tell to an emotional experience of architecture via a functional MR study? | Frontiers of Architectural Research | 2022 | 10.1016/j.foar.2022.02.007 |
| 212 | Night heart rate variability and particulate exposures among boilermaker construction workers | Environmental Health Perspectives | 2007 | 10.1289/ehp.10019 |
| 213 | NIR Scaffold Bearing Three Handles for Biocompatible Sequential Click Installation of Multiple Functional Arms | Journal of The American Chemical Society | 2020 | 10.1021/jacs.9b10467 |
| 214 | Noise Reference Signal-Based Denoising Method for EDA Collected by Multimodal Biosensor Wearable in the Field | Journal of Computing in Civil Engineering | 2020 | 10.1061/(ASCE)CP.1943-5487.0000927 |
| 215 | Normative Visual Patterns for Hazard Recognition: A Crisp-Set Qualitative Comparative Analysis Approach | Ksce Journal of Civil Engineering | 2021 | 10.1007/s12205-021-1362-5 |
| 216 | Occupant behavior long-term continuous monitoring integrated to prediction models: Impact on office building energy performance | Energy | 2019 | 10.1016/j.energy.2019.04.005 |
| 217 | Older workers in the construction industry: Results of a routine health examination and a five year follow up | Occupational and Environmental Medicine | 1996 | 10.1136/oem.53.10.686 |
| 218 | Overcoming Status Quo Bias for Resilient Stormwater Infrastructure: Empirical Evidence in Neurocognition and Decision-Making | Journal of Management in Engineering | 2020 | 10.1061/(ASCE)ME.1943-5479.0000771 |
| 219 | Participatory intervention with objectively measured physical risk factors for musculoskeletal disorders in the construction industry: study protocol for a cluster randomized controlled trial | Bmc Musculoskeletal Disorders | 2015 | 10.1186/s12891-015-0758-0 |
| 220 | Paving the Way for Future EEG Studies in Construction: Dependent Component Analysis for Automatic Ocular Artifact Removal from Brainwave Signals | Journal of Construction Engineering and Management | 2021 | 10.1061/(ASCE)CO.1943-7862.0002097 |
| 221 | Physical capacity, occupational physical demands, and relative physical strain of older employees in construction and healthcare | international Archives of Occupational and Environmental Health | 2019 | 10.1007/s00420-018-1377-5 |
| 222 | Physiological activity in calm thermal indoor environments | Scientific Reports | 2017 | 10.1038/s41598-017-11755-3 |
| 223 | Physiological and cognitive performance of exposure to biophilic indoor environment | Building and Environment | 2018 | 10.1016/j.buildenv.2018.01.006 |
| 224 | Physiological and subjective comfort evaluation under different airflow directions in a cooling environment | Plos One | 2021 | 10.1371/journal.pone.0249235 |
| 225 | Physiological effects of blue pulsed led in an indoor lighting environment | Revista Mexicana De ingenieria Biomedica | 2018 | 10.17488/RMIB.39.3.6 |
| 226 | Physiological responses to elevated carbon dioxide concentration and mental workload during performing MATB tasks | Building and Environment | 2021 | 10.1016/j.buildenv.2021.107752 |
| 227 | Pilot study of the effects of ventilation and ventilation noise on sleep quality in the young and elderly | indoor Air | 2021 | 10.1111/ina.12861 |
| 228 | PM2.5 metal exposures and nocturnal heart rate variability: a panel study of boilermaker construction workers | Environmental Health | 2008 | 10.1186/1476-069X-7-36 |
| 229 | Predicting workers' inattentiveness to struck-by hazards by monitoring biosignals during a construction task: A virtual reality experiment | Advanced Engineering informatics | 2021 | 10.1016/j.aei.2021.101359 |
| 230 | Prediction of Human Performance Using Electroencephalography under Different Indoor Room Temperatures | Brain Sciences | 2018 | 10.3390/brainsci8040074 |
| 231 | Pre-service fatigue screening for construction workers through wearable EEG-based signal spectral analysis | Automation in Construction | 2019 | 10.1016/j.autcon.2019.102851 |
| 232 | Proactive Safety Measures: Quantifying the Upright Standing Stability after Sustained Rebar Tying Postures | Journal of Construction Engineering and Management | 2018 | 10.1061/(ASCE)CO.1943-7862.0001458 |
| 233 | Psychological and neural responses to architectural interiors | Cortex | 2020 | 10.1016/j.cortex.2020.01.009 |
| 234 | Psychological and physiological effects of a green wall on occupants: A cross-over study in virtual reality | Building and Environment | 2021 | 10.1016/j.buildenv.2021.108134 |
| 235 | Psycho-physiological responses to plant variegation as measured through eye movement, self-reported emotion and cerebral activity | indoor and Built Environment | 2017 | 10.1177/1420326X16638711 |
| 236 | Quantifying Human Experience in Architectural Spaces with Integrated Virtual Reality and Body Sensor Networks | Journal of Computing in Civil Engineering | 2019 | 10.1061/(ASCE)CP.1943-5487.0000812 |
| 237 | Re-assessing hazard recognition ability in occupational environment with microvascular function in the brain | Safety Science | 2019 | 10.1016/j.ssci.2019.06.040 |
| 238 | Reinvestigation of the Psychological Mechanisms of Construction Experience on Hazard Recognition Performance | Human Factors | 2022 | 10.1177/00187208211066666 |
| 239 | Relationship between discomfort glare and electroencephalography during working in front of a window | Journal of Environmental Engineering (Japan) | 2021 | 10.3130/aije.86.121 |
| 240 | Relationship between rework of engineering drawing tasks and stress level measured from physiological signals | Automation in Construction | 2021 | 10.1016/j.autcon.2021.103560 |
| 241 | Research on electroencephalogram to measure thermal pleasure in thermal alliesthesia in temperature step-change environment | indoor Air | 2018 | 10.1111/ina.12491 |
| 242 | Research on environmental comfort and cognitive performance based on EEG plus VR plus LEC evaluation method in underground space | Building and Environment | 2021 | 10.1016/j.buildenv.2021.107886 |
| 243 | Research on Generating an Indoor Landmark Salience Model for Self-Location and Spatial Orientation from Eye-Tracking Data | Isprs international Journal of Geo-information | 2020 | 10.3390/ijgi9020097 |
| 244 | Research on the light comfort characterization method based on visual evoked potential energy | Building and Environment | 2021 | 10.1016/j.buildenv.2021.107831 |
| 245 | Research on work efficiency and light comfort based on EEG evaluation method | Building and Environment | 2020 | 10.1016/j.buildenv.2020.107122 |
| 246 | Research Trends of Human-Computer Interaction Studies in Construction Hazard Recognition: A Bibliometric Review | Sensors | 2021 | 10.3390/s21186172 |
| 247 | Restorative benefits of urban green space: Physiological, psychological restoration and eye movement analysis | Journal of Environmental Management | 2022 | 10.1016/j.jenvman.2021.113930 |
| 248 | Revealing the Invisible Gorilla in construction: Estimating construction safety through mental workload assessment | Automation in Construction | 2016 | 10.1016/j.autcon.2015.12.018 |
| 249 | Revealing the relationships between luminous environment characteristics and physiological, ocular and performance measures: An experimental study | Building and Environment | 2020 | 10.1016/j.buildenv.2020.106702 |
| 250 | Review visual attention and spatial memory in building inspection: Toward a cognition-driven information system | Advanced Engineering informatics | 2020 | 10.1016/j.aei.2020.101061 |
| 251 | Role of Personality in Construction Safety: Investigating the Relationships between Personality, Attentional Failure, and Hazard Identification under Fall-Hazard Conditions | Journal of Construction Engineering and Management | 2019 | 10.1061/(ASCE)CO.1943-7862.0001673 |
| 252 | Scoping Review of EEG Studies in Construction Safety | international Journal of Environmental Research and Public Health | 2019 | 10.3390/ijerph16214146 |
| 253 | Seeing the city: using eye-tracking technology to explore cognitive responses to the built environment | Journal of Urbanism | 2019 | 10.1080/17549175.2018.1531908 |
| 254 | Selection of wearable sensor measurements for monitoring and managing entry-level construction worker fatigue: a logistic regression approach | Engineering Construction and Architectural Management | 2021 | 10.1108/ECAM-02-2021-0106 |
| 255 | Sex differences in muscular load among house painters performing identical work tasks | European Journal of Applied Physiology | 2014 | 10.1007/s00421-014-2918-6 |
| 256 | Short- and long-term effects of architecture on the brain: Toward theoretical formalization | Frontiers of Architectural Research | 2019 | 10.1016/j.foar.2019.07.004 |
| 257 | Simulation-Based Assessment of Workers' Muscle Fatigue and Its Impact on Construction Operations | Journal of Construction Engineering and Management | 2016 | 10.1061/(ASCE)CO.1943-7862.0001182 |
| 258 | Studies on visual health features of luminous environment in college classrooms | Building and Environment | 2021 | 10.1016/j.buildenv.2021.108184 |
| 259 | Study of Multidimensional Design Approaches to Emergency Signage | international Journal of Asia Digital Art and Design | 2021 | 10.20668/adada.25.3_39 |
| 260 | Study on Environmental Parameters and Wearing Electrocardiogram | Sensors and Materials | 2019 | 10.18494/SAM.2019.2223 |
| 261 | Study on the variation of peak isometric strength and EMG activity in static field-simulated lifting postures | international Journal of industrial Ergonomics | 2004 | 10.1016/j.ergon.2003.07.002 |
| 262 | Studying the impact of built environments on human mental health in everyday life: methodological developments, state-of-the-art and technological frontiers | Current Opinion in Psychology | 2020 | 10.1016/j.copsyc.2019.08.026 |
| 263 | Subjective and physiological responses to facade and sunlight pattern geometry in virtual reality | Building and Environment | 2019 | 10.1016/j.buildenv.2019.01.009 |
| 264 | Subjective experience and visual attention to a historic building: A real-world eye-tracking study | Frontiers of Architectural Research | 2020 | 10.1016/j.foar.2020.07.006 |
| 265 | Suitability of Eye Tracking in Assessing the Visual Perception of Architecture-A Case Study Concerning Selected Projects Located in Cologne | Buildings | 2020 | 10.3390/buildings10020020 |
| 266 | Supervised machine learning of thermal comfort under different indoor temperatures using EEG measurements | Energy and Buildings | 2020 | 10.1016/j.enbuild.2020.110305 |
| 267 | Taking a closer look at indoor route guidance; usability study to compare an adapted and non-adapted mobile prototype | Spatial Cognition and Computation | 2021 | 10.1080/13875868.2021.1885411 |
| 268 | Temporal Visual Patterns of Construction Hazard Recognition Strategies | international Journal of Environmental Research and Public Health | 2021 | 10.3390/ijerph18168779 |
| 269 | The effect of a knee support on the biomechanical response of the low back | Journal of Applied Biomechanics | 2007 | 10.1123/jab.23.4.275 |
| 270 | The effect of dynamic correlated colour temperature changes on alertness and performance | Lighting Research & Technology | 2018 | 10.1177/1477153518755617 |
| 271 | The effect of indoor plants on human comfort | indoor and Built Environment | 2014 | 10.1177/1420326X13481372 |
| 272 | The Effect of Lift Teams on Kinematics and Muscle Activity of the Upper Extremity and Trunk in Bricklayers | Journal of Orthopaedic & Sports Physical Therapy | 2013 | 10.2519/jospt.2013.4249 |
| 273 | The effect of noise content and level on cognitive performance measured by electroencephalography (EEG) | Automation in Construction | 2021 | 10.1016/j.autcon.2021.103836 |
| 274 | The effectiveness of evacuation signs in buildings based on eye tracking experiment | Natural Hazards | 2020 | 10.1007/s11069-020-04030-8 |
| 275 | The effects of air temperature on office workers' well-being, workload and productivity-evaluated with subjective ratings | Applied Ergonomics | 2010 | 10.1016/j.apergo.2010.04.003 |
| 276 | The effects of higher temperature setpoints during summer on office workers' cognitive load and thermal comfort | Building and Environment | 2017 | 10.1016/j.buildenv.2017.06.048 |
| 277 | The effects of indoor plants and artificial windows in an underground environment | Building and Environment | 2018 | 10.1016/j.buildenv.2018.04.029 |
| 278 | The effects of interior design on wellness - Eye tracking analysis in determining emotional experience of architectural space. A survey on a group of volunteers from the Lublin Region, Eastern Poland | Annals of Agricultural and Environmental Medicine | 2020 | 10.26444/aaem/106233 |
| 279 | The effects of intermittent light during the evening on sleepiness, sleep electroencephalographic spectral power and performance the next morning | Lighting Research & Technology | 2019 | 10.1177/1477153519828414 |
| 280 | The effects of sound loudness on subjective feeling, sympathovagal balance and brain activity | indoor and Built Environment | 2018 | 10.1177/1420326X17719490 |
| 281 | The impact of engineering information format on task performance: Gaze scanning pattern analysis | Advanced Engineering informatics | 2020 | 10.1016/j.aei.2020.101167 |
| 282 | The impact of engineering information formats on learning and execution of construction operations: A virtual reality pipe maintenance experiment | Automation in Construction | 2020 | 10.1016/j.autcon.2020.103367 |
| 283 | The impact of indoor air temperature on the executive functions of human brain and the physiological responses of body | Health Promotion Perspectives | 2019 | 10.15171/hpp.2019.07 |
| 284 | The neuroaesthetics of architectural spaces | Cognitive Processing | 2021 | 10.1007/s10339-021-01043-4 |
| 285 | The practice and potential of heritage emotion research: an experimental mixed-methods approach to investigating affect and emotion in a historic house | international Journal of Heritage Studies | 2020 | 10.1080/13527258.2020.1714696 |
| 286 | The Role of Expertise in Visual Exploration and Aesthetic Judgment of Residential Building Facades: An Eye-Tracking Study | Psychology of Aesthetics Creativity and The Arts | 2021 | 10.1037/aca0000377 |
| 287 | The Role of Symmetry in the Aesthetics of Residential Building Facades Using Cognitive Science Methods | Symmetry-Basel | 2020 | 10.3390/sym12091438 |
| 288 | Unannounced Evacuation Experiment in a High-Rise Hotel Building with Evacuation Elevators: A Study of Evacuation Behaviour Using Eye-Tracking | Fire Technology | 2021 | 10.1007/s10694-020-01046-1 |
| 289 | Use of biometric data and EEG to assess architectural quality of two office spaces: a pilot experiment | intelligent Buildings international | 2021 | 10.1080/17508975.2021.1921683 |
| 290 | Use of electroencephalogram and long short-term memory networks to recognize design preferences of users toward architectural design alternatives | Journal of Computational Design and Engineering | 2020 | 10.1093/jcde/qwaa045 |
| 291 | Use of electroencephalography (EEG) for comparing study of the external space perception of traditional and modern commercial districts | Journal of Asian Architecture and Building Engineering | 2021 | 10.1080/13467581.2020.1813586 |
| 292 | Using an eye tracker to study three-dimensional environmental aesthetics: the impact of architectural elements and educational training on viewers' visual attention | Journal of Architectural and Planning Research | 2015 | Null |
| 293 | Using electroencephalogram to continuously discriminate feelings of personal thermal comfort between uncomfortably hot and comfortable environments | indoor Air | 2020 | 10.1111/ina.12644 |
| 294 | Using eye-tracker to compare search patterns between experienced and novice workers for site hazard identification | Safety Science | 2016 | 10.1016/j.ssci.2015.08.008 |
| 295 | Using Eye-Tracking to Understand Human Responses to Traditional Neighborhood Designs | Planning Practice and Research | 2020 | 10.1080/02697459.2020.1768332 |
| 296 | Using Posterior EEG Theta Band to Assess the Effects of Architectural Designs on Landmark Recognition in an Urban Setting | Frontiers in Human Neuroscience | 2020 | 10.3389/fnhum.2020.584385 |
| 297 | Using Ryodoraku Measurement to Evaluate the Impact of Environmental Noise on Human Physiological Response | indoor and Built Environment | 2012 | 10.1177/1420326X11409448 |
| 298 | Visual Attention Software: A New Tool for Understanding the Subliminal Experience of the Built Environment | Applied Sciences-Basel | 2021 | 10.3390/app11136197 |
| 299 | Visual fatigue following long-term visual display terminal work under different light sources | Lighting Research & Technology | 2017 | 10.1177/1477153516677559 |
| 300 | Walking through Architectural Spaces: The Impact of Interior Forms on Human Brain Dynamics | Frontiers in Human Neuroscience | 2017 | 10.3389/fnhum.2017.00477 |
| 301 | Wayfinding Information Cognitive Load Classification Based on Functional Near-Infrared Spectroscopy | Journal of Computing in Civil Engineering | 2021 | 10.1061/(ASCE)CP.1943-5487.0000984 |
| 302 | Wayfunding in aging and Alzheimer's disease within a virtual senior residence: study protocol | Journal of Advanced Nursing | 2016 | 10.1111/jan.12945 |
| 303 | Wearable Sensing Technology Applications in Construction Safety and Health | Journal of Construction Engineering and Management | 2019 | 10.1061/(ASCE)CO.1943-7862.0001708 |
| 304 | What part of the brain is involved in graphic design thinking in landscape architecture? | Plos One | 2021 | 10.1371/journal.pone.0258413 |
| 305 | Where do we look when we walk on stairs? Gaze behaviour on stairs, transitions, and handrails | Experimental Brain Research | 2011 | 10.1007/s00221-010-2520-z |
| 306 | Window view and relaxation: Viewing green space from a high-rise estate improves urban dwellers' wellbeing | Urban Forestry & Urban Greening | 2020 | 10.1016/j.ufug.2020.126846 |
| 307 | Window View and the Brain: Effects of Floor Level and Green Cover on the Alpha and Beta Rhythms in a Passive Exposure EEG Experiment | international Journal of Environmental Research and Public Health | 2018 | 10.3390/ijerph15112358 |
